# The Effect of India's Total Sanitation Campaign on Defecation Behaviors and Child Health in Rural Madhya Pradesh: A Cluster Randomized Controlled Trial

**DOI:** 10.1371/journal.pmed.1001709

**Published:** 2014-08-26

**Authors:** Sumeet R. Patil, Benjamin F. Arnold, Alicia L. Salvatore, Bertha Briceno, Sandipan Ganguly, John M. Colford, Paul J. Gertler

**Affiliations:** 1 Network for Engineering and Economics Research and Management (NEERMAN), Mumbai, Maharashtra, India; 2 School of Public Health, University of California, Berkeley, California, United States of America; 3 Stanford School of Medicine, Stanford University, Stanford, California, United States of America; 4 Water and Sanitation Program, the World Bank, Washington (D.C.), United States of America; 5 National Institute for Cholera and Enteric Diseases, Kolkata, West Bengal, India; 6 Haas School of Business, University of California, Berkeley, California, United States of America; University of East Anglia, United Kingdom

## Abstract

**Background:**

Poor sanitation is thought to be a major cause of enteric infections among young children. However, there are no previously published randomized trials to measure the health impacts of large-scale sanitation programs. India's Total Sanitation Campaign (TSC) is one such program that seeks to end the practice of open defecation by changing social norms and behaviors, and providing technical support and financial subsidies. The objective of this study was to measure the effect of the TSC implemented with capacity building support from the World Bank's Water and Sanitation Program in Madhya Pradesh on availability of individual household latrines (IHLs), defecation behaviors, and child health (diarrhea, highly credible gastrointestinal illness [HCGI], parasitic infections, anemia, growth).

**Methods and Findings:**

We conducted a cluster-randomized, controlled trial in 80 rural villages. Field staff collected baseline measures of sanitation conditions, behaviors, and child health (May–July 2009), and revisited households 21 months later (February–April 2011) after the program was delivered. The study enrolled a random sample of 5,209 children <5 years old from 3,039 households that had at least one child <24 months at the beginning of the study. A random subsample of 1,150 children <24 months at enrollment were tested for soil transmitted helminth and protozoan infections in stool. The randomization successfully balanced intervention and control groups, and we estimated differences between groups in an intention to treat analysis. The intervention increased percentage of households in a village with improved sanitation facilities as defined by the WHO/UNICEF Joint Monitoring Programme by an average of 19% (95% CI for difference: 12%–26%; group means: 22% control versus 41% intervention), decreased open defecation among adults by an average of 10% (95% CI for difference: 4%–15%; group means: 73% intervention versus 84% control). However, the intervention did not improve child health measured in terms of multiple health outcomes (diarrhea, HCGI, helminth infections, anemia, growth). Limitations of the study included a relatively short follow-up period following implementation, evidence for contamination in ten of the 40 control villages, and bias possible in self-reported outcomes for diarrhea, HCGI, and open defecation behaviors.

**Conclusions:**

The intervention led to modest increases in availability of IHLs and even more modest reductions in open defecation. These improvements were insufficient to improve child health outcomes (diarrhea, HCGI, parasite infection, anemia, growth). The results underscore the difficulty of achieving adequately large improvements in sanitation levels to deliver expected health benefits within large-scale rural sanitation programs.

**Trial Registration:**

ClinicalTrials.gov NCT01465204

*Please see later in the article for the Editors' Summary*

## Abstract 2

Sumeet Patil and colleagues conduct a cluster randomized controlled trial to measure the effect of India's Total Sanitation Campaign in Madhya Pradesh on the availability of individual household latrines, defecation behaviors, and child health.

*Please see later in the article for the Editors' Summary*

## Editors' Summary

**Background:**

Diarrheal diseases are linked with the deaths of hundreds of thousands of young children each year in resource-limited countries. Infection with enteric pathogens (organisms such as bacteria, viruses, and parasites that infect the human intestine or gut) also affects the health and growth of many young children in these countries. A major contributor to the transmission of enteric pathogens is thought to be open defecation, which can expose individuals to direct contact with human feces containing infectious pathogens and also contaminate food and drinking water. Open defecation can be reduced by ensuring that people have access to and use toilets or latrines. Consequently, programs have been initiated in many resource-limited countries that aim to reduce open defecation by changing behaviors and by providing technical and financial support to help households build improved latrines (facilities that prevent human feces from re-entering the environment such as pit latrines with sealed squat plates; an example of an unimproved facility is a simple open hole). However, in 2011, according to the WHO/UNICEF Joint Monitoring Programme for Water Supply and Sanitation, more than 1 billion people (15% of the global population) still defecated in the open.

**Why Was This Study Done?:**

Studies of sewerage system provision in urban areas suggest that interventions that prevent human feces entering the environment reduce diarrheal diseases. However, little is known about how rural sanitation programs, which usually focus on providing stand-alone sanitation facilities, affect diarrheal disease, intestinal parasite infections, anemia (which can be caused by parasite infections), or growth in young children. Governments and international donors need to know whether large-scale rural sanitation programs improve child health before expending further resources on these interventions or to identify an urgency to improve the existing program design or implementation so that they deliver the health impact. In this study, the researchers investigate the effect of India's Total Sanitation Campaign (TSC) on the availability of individual household latrines, defecation behaviors, and child health in rural Madhya Pradesh, one of India's less developed states. Sixty percent of people who practice open defection live in India and a quarter of global child deaths from diarrheal diseases occur in the country. India's TSC, which was initiated in 1999, includes activities designed to change social norms and behaviors and provides technical and financial support for latrine building. So far there are no published studies that rigorously evaluated whether the TSC improved child health or not.

**What Did the Researchers Do and Find?:**

A cluster randomized controlled trial randomly assigns groups of people to receive the intervention under study and compares the outcomes with a control group that does not receive the intervention. The researchers enrolled 5,209 children aged under 5 years old living in 3,039 households in 80 rural villages in Madhya Pradesh. Half of the villages (40), chosen at random, were included in the TSC (the intervention). Field staff collected data on sanitation conditions, defecation behaviors, and child health from caregivers in each household at the start of the study and after the TSC implementation was over in the intervention villages. A random subsample of children was also tested for infection with enteric parasites. The intervention increased the percentage of households in a village with improved sanitation facilities by 19% on average. Specifically, 41% of households in the intervention villages had improved latrines on average compared to 22% of households in the control villages. The intervention also decreased the proportion of adults who self-reported open defecation from 84% to 73%. However, the intervention did not improve child health measured on the basis of multiple health outcomes, including the prevalence of gastrointestinal illnesses and intestinal parasite infections, and growth.

**What Do These Findings Mean?:**

These findings indicate that in rural Madhya Pradesh, the TSC implemented with support from the WSP only slightly increased the availability of individual household latrines and only slightly decreased the practice of open defecation. Importantly, these findings show that these modest improvements in sanitation and in defecation behaviors were insufficient to improve health outcomes among children. The accuracy of these findings may be limited by various aspects of the study. For example, several control villages actually received the intervention, which means that these findings probably underestimate the effect of the intervention under perfect conditions. Self-reporting of defecation behavior, availability of sanitation facilities, and gastrointestinal illnesses among children may also have biased these findings. Finally, because TSC implementation varies widely across India, these findings may not apply to other Indian states or variations in the TSC implementation strategies. Overall, however, these findings highlight the challenges associated with achieving large enough improvement in access to sanitation and correspondingly large reductions in the practice of open defecation to deliver health benefits within large-scale rural sanitation programs.

**Additional Information:**

Please access these websites via the online version of this summary at http://dx.doi.org/10.1371/journal.pmed.1001709.

- This study is further discussed in a *PLOS Medicine* Perspective by Clarissa Brocklehurst
- A *PLOS Medicine* Collection on water and sanitation is available
- The World Health Organization (WHO) provides information on water, sanitation, and health (in several languages), on diarrhea (in several languages), and on intestinal parasites (accessed through WHO's web page on neglected tropical diseases); the 2009 WHO/UNICEF report “Diarrhea: why children are still dying and what can be done”, is available online for download
- The WHO/UNICEF Joint Monitoring Programme for Water Supply and Sanitation monitors progress toward improved global sanitation; its 2014 update report is available online
- The children's charity UNICEF, which protects the rights of children and young people around the world, provides information on water, sanitation, and hygiene, and on diarrhea (in several languages)

## Introduction

The practice of open defecation is thought to be a major cause of the persistent worldwide burden of diarrhea and enteric parasite infection among children <5 years old [1]. Reducing open defecation requires access to and use of improved sanitation facilities, which are defined as facilities that prevent human feces from re-entering the environment [2]. In 2010, an estimated 47% of the world's population did not have access to improved sanitation facilities. India alone accounts for a third of those without improved sanitation (814 million), nearly 60% of those who practice open defecation (626 million) [2], and a quarter of the world's deaths from diarrheal diseases among children aged less than 5 years [3].

Observational studies of interventions that prevent human feces from entering the environment have been shown to reduce diarrheal diseases [4],[5] and enteric parasite infections [6]–[8]. Most of this research, however, has focused on the provision of sewerage systems in urban centers. Few studies have been conducted in rural areas of low-income countries where the provision and maintenance of networked sewerage is prohibitively expensive. Consequently, most government and donor financing in the rural sanitation sector focuses on the provision of non-networked toilets. Despite the wide scale deployment of such programs, to our knowledge there have been no published randomized trials to measure the effect of rural sanitation programs on diarrheal diseases, intestinal parasite infections, anemia, or growth in young children.

The objective of this study was to measure the effect of India's Total Sanitation Campaign (TSC) in rural Madhya Pradesh on household availability of improved sanitation facilities as defined by WHO/UNICEF Joint Monitoring Programme (JMP) for water and sanitation [2], open defecation behaviors of household members, water quality, and child health (diarrheal diseases, highly credible gastrointestinal illness [HCGI], enteric parasite infections, anemia, and growth). The TSC, scaled up to all districts in India and deployed to hundreds of millions of people, is possibly the largest rural sanitation program in the world. As a part of their Total Sanitation and Sanitation Marketing (TSSM) project, the Water and Sanitation Program (WSP; the World Bank) provided capacity building support to ten districts of Madhya Pradesh to strengthen the implementation of the program. In two of these ten districts, we studied the effects of the TSC implemented with support from the WSP under the TSSM project using a cluster-randomized controlled trial in 80 rural villages.

**Figure 1:**
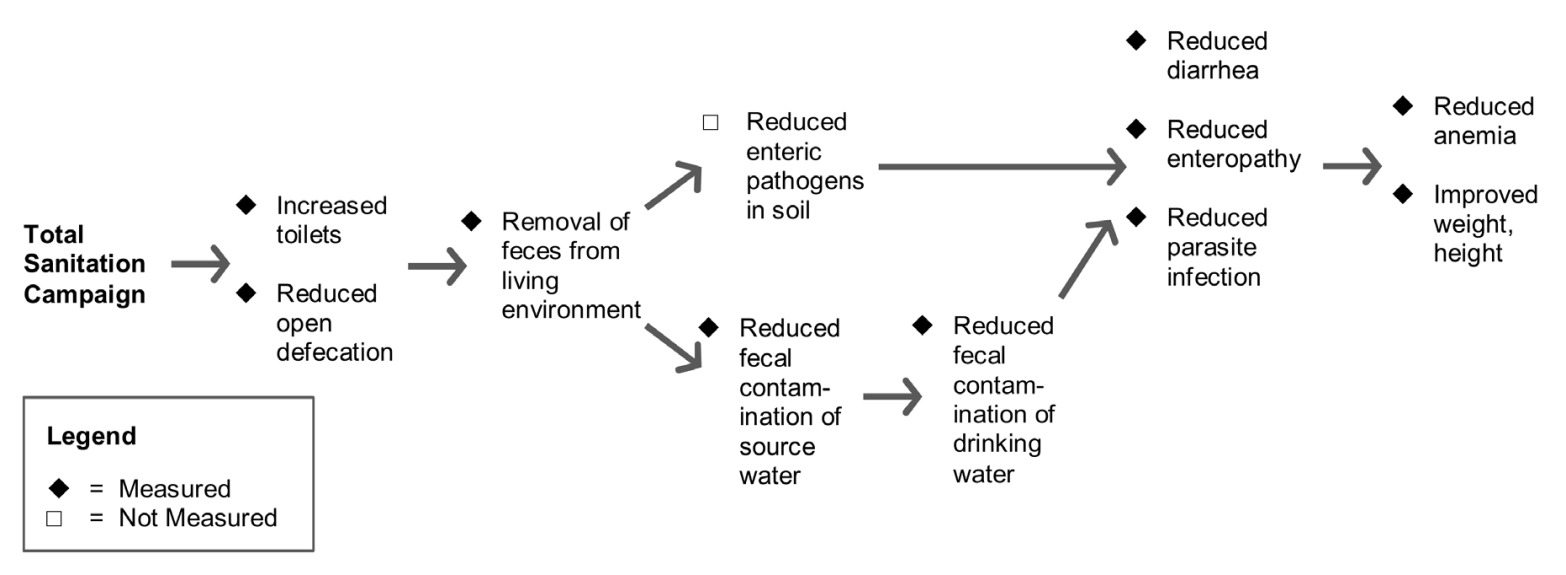
Hypothesized causal pathways for intervention impact and measurements.

We hypothesized that the program would increase availability of individual household latrines (IHLs) and reduce the practice of open defecation in a community through use of IHLs. On the basis of previous research [4]–[8], we further hypothesized that less open defecation would: (i) reduce the quantity of feces in the environment that could contaminate shallow groundwater aquifers, water distribution networks, and soil in the community, and (ii) also reduce enteric pathogen transmission through flies, which are well-established vectors for transmission [9]–[11]. Conditional on improvements in these intermediate outcomes, we hypothesized that children <24 months at enrollment in intervention villages would have a lower prevalence of diarrhea, HCGI, enteric parasite infections, and anemia when measured after the intervention. Finally, we hypothesized that the program would improve average weight-for-age and height-for-age in these young children as a result of fewer symptomatic and asymptomatic enteric infections over longer exposure periods to improved sanitation [12]–[16]. The above hypothesized causal chain between the intervention and health outcomes is depicted in Figure 1.

## Methods

### Ethics Statement

The study is a part of larger six-country study commissioned by the WSP. The study protocol was approved by the Western Institutional Review Board, Olympia, Washington, USA (study number 1095420) and the Independent Ethics Committee, Mumbai, India (IEC/09/11). The survey respondents provided a verbal consent after enumerators apprised them of study objectives, use of collected information, confidentiality, risks, benefits, and respondent rights. Written consent was not obtained because of lower literacy and social norms that would deter women (child caregivers were the main respondents) from signing any document without her husband's or elders' permission.

The protocol for the broader study was formally registered after the completion of fieldwork because at the time the study was conceived pre-registration was not a well-known convention in the field of development economics [17] (the study was originally conceived by PJG and colleagues at the World Bank). The team agreed that a late registration was better than none at all. The original study protocol—established in 2008; before the baseline survey—included another two districts from the state of Himachal Pradesh where the WSP provided support under their TSSM project as well [18]. However, the study in Himachal Pradesh was discontinued because it was impossible to retain a control group for the duration of the study period. The only substantive change to the protocol in Madhya Pradesh after the start of the trial was the increase in sample size planned for follow-up; we provide details in the section on Sample Size. The CONSORT checklist (Text S1) and the follow-up study protocol (Text S2) are provided as supplemental information.

### Trial Design

The study design was a cluster randomized controlled trial with randomization at the village level and equal allocation to the two treatment arms. The study population included 80 villages from two neighboring districts in Madhya Pradesh: Dhar and Khargone. The villages randomized to the intervention group received the TSC program and villages in the control group did not receive the TSC until after the study. As a demand driven program, the district administration was duty bound to provide the program to the villages in the control group if they requested it and if the funding was available. The district administration agreed to provide the program to all control villages after the completion of the study. The study measured outcomes, anticipated confounders, and covariates at household and child levels both before and after the intervention in two survey waves. The follow-up survey was administered to the same households who participated in baseline data collection and additional households were included at follow-up (see the section on Sample Size for details).

### Study Population

Table 1 describes population characteristics for the study region relative to the state and national population on the basis of India's 2011 Census. Overall, Madhya Pradesh is one of the less developed states of India, including its water and sanitation infrastructure. The study districts are more agricultural, with higher proportion of marginalized population groups and lower literacy than the state average, but with better water supply and drainage infrastructure. IHL coverage (percentage of households with access to IHL) in rural areas of study districts (19.2% in Dhar and 13% in Khargone) is comparable to the state average (13.1%) but much worse than the country average (30.7%). The IHLs are predominantly the types included in the JMP definition of improved sanitation [2]. On average the IHL coverage across India increased by approximately 10% between 2001 and the 2011 Census. However, the change in the IHL coverage between 2001 and 2011 varied widely between states and between districts within each state [19].

**Table 1:** Descriptive statistics for India, Madhya Pradesh, and study districts, Census 2011.

Indicators	India	Madhya Pradesh	Dhar District	Khargone District
**Population and occupations**				
Total population	1,210,569,573	72,626,809	2,185,793	1,873,046
Rural population	833,463,448	52,557,404	1,772,572	1,574,190
Percent rural population	68.80	72.40	81.10	84.00
Percent 0–6 years children (of rural population)	14.60	15.80	16.90	16.60
Percent SCST (of rural population)	29.70	42.90	70.60	55.80
Percent literates (of >6 years rural population)	67.80	63.90	54.10	58.90
Percent of cultivators (of rural workers)	33.00	38.30	42.90	38.30
Percent of agriculture laborers (of rural workers)	39.30	47.30	47.70	52.20
Percent of other occupations (of rural workers)	27.70	14.40	9.40	9.50
**Water and sanitation**				
Number of rural households (RHHs)	167,826,730	11,122,365	339,844	309,603
Percent RHHs with permanent/good house construction	45.90	33.40	38.90	31.50
Percent RHHs with improved drinking water source^a^	84.30	74.10	79.90	84.20
Percent RHHs with access to tap water (on premise or away)	30.80	9.90	19.70	41.10
Percent RHHs with on premise water source (any type)	35.00	13.00	13.50	24.60
Percent RHHs with bathing rooms	45.00	34.00	38.10	50.40
Percent RHHs with closed drainage	5.70	2.10	3.20	4.20
Percent RHHs with open drainage	31.00	23.10	24.00	43.30
**Latrine availability**				
Percent RHHs with on-premise latrine^b^	30.73	13.12	19.17	13.00
Flush toilet connected to piped sewer system	2.20	0.80	1.43	1.15
Flush toilet connected to septic tank	14.70	8.32	12.91	9.07
Flush toilet connected to other system	2.53	1.26	1.25	0.70
Pit latrine with slab/ventilated improved pit	8.19	1.79	2.23	1.50
Pit latrine without slab/open pit	2.34	0.76	1.12	0.41
Toilets disposing waste to open drain	0.22	0.10	0.13	0.07
Serviced toilets where waste is removed by humans	0.35	0.03	0.03	0.02
Serviced toilets where waste is removed by animals	0.19	0.07	0.06	0.08
Percent RHHs with access to public toilets	1.94	0.46	0.68	0.36
Percent RHHs with no toilet/open site (2011)	67.33	86.42	80.15	86.65
Percent RHHs with no toilet/open site (2001)	78.10	91.10	86.40	91.10

a Improved drinking water sources include tap water, covered well, hand pump, and tube well as defined by Census of India, 2011.

b On premise latrines are also referred to as IHLs. The first four types of toilets—flush toilets connected to sewer system, septic tank or other systems, and pit latrine slab and/or ventilated improved pit—are a subset of latrine types included in the definition of improved sanitation by WHO/UNICEF JMP for water and sanitation [2].

SCST, Schedule Caste or Schedule Tribe (marginalized population group); RHH, rural household.

Study villages were selected in collaboration with the Madhya Pradesh state government. Madhya Pradesh is divided into 50 districts, 313 Blocks, and 23,040 *Gram Panchayats* (referred to as “villages” in this manuscript). A *Gram Panchayat* is the smallest Indian administrative unit and has a local elected body. The 80 study villages were the independent units selected in three steps. First, through a series of meetings and site visits, the state government and the WSP selected two of 50 districts in Madhya Pradesh: Dhar and Khargone. Second, 11 of 13 Blocks from Dhar and eight of nine Blocks from Khargone were selected for the study. The remaining Blocks were excluded from the sample frame because all villages from these Blocks were earmarked for the TSC program, precluding the enrollment of control villages. Third, in each administrative Block the government identified villages where they were amendable to randomizing the TSC program.

**Figure 2:**
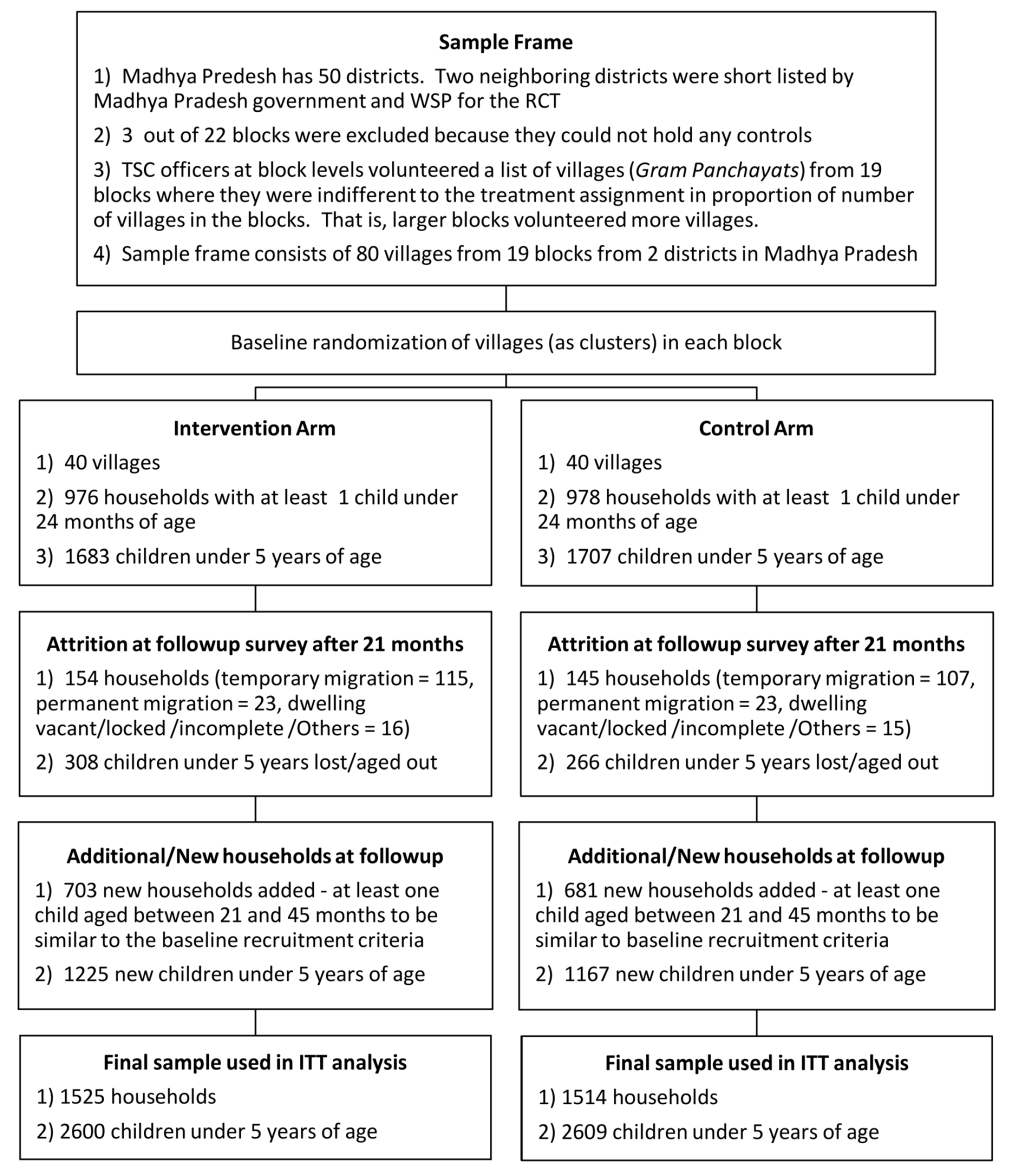
CONSORT Flowchart: enrollment, intervention allocation, attrition, and addition of participants.

In each of the 80 study villages, the field team listed and mapped 200 households and randomly selected 25 households with at least one child <24 months of age at enrollment. If a village had multiple sub-villages, then to avoid spreading the sample too thin, the survey team selected the most populous two to three sub-villages for the listing purposes. From the numbered list of eligible households, a random starting number was chosen and thereafter every *n* ^th^ household number was selected where *n* was determined by dividing eligible number of households by 25. For the follow-up survey we increased the sample size of households per village from 25 to 38 (see section on Sample Size). Additional 100 to 150 households were listed and mapped before the follow-up survey to select additional households. Figure 2 summarizes loss to follow-up in the original cohort and recruitment of new households in the follow-up survey. Because we conducted the follow-up survey 21 months after baseline, the eligibility criteria for newly enrolled households was that they had at least one child between the ages of 21 months and 45 months and were living in the village at the time of the baseline survey to be commensurate with the eligibility criteria for the original cohort. Child caregivers were the main survey respondents, but household heads or other elders occasionally answered questions related to household characteristics.

### Intervention Program

India's TSC, initiated in 1999, was an ambitious program with a goal to eliminate the practice of open defecation in India by 2012. In 2012, the government transformed TSC into a new program named *Nirmal Bharat Abhiyaan* (Clean India Campaign). The TSC included subsidies for and promotion of IHLs that can safely confine feces (similar to JMP defined improved sanitation facilities), school sanitation and hygiene education, *Anganwadi* (preschool) toilets, and community sanitation complexes. The TSC also supported rural sanitary marts and production centers to provide good quality but affordable material for toilet construction. Additionally, the TSC included several features such as ongoing social mobilization and behavior change activities at state, district, and village levels, flexible technology options for toilets, and a community award called the *Nirmal Gram Puraskar* (NGP) given to communities that were determined to be “open defecation free”—defined as a community where all households have and use IHLs that can safely confine feces—and meet all of the other “total sanitation” requirements defined by the Indian government. The NGP awards ranged from Rs 50,000 (US$1,000) to Rs 500,000 (US$10,000) for villages, up to Rs 2,000,000 (US$40,000) for Blocks, and Rs 5,000,000 (US$100,000) for districts.

In Madhya Pradesh, the TSC was implemented with a concurrent program named *Nirmal Vatika* (Clean House) under the National Rural Employment Guarantee Scheme to provide additional financial and material subsidies to households. TSC and *Nirmal Vatika* together provided at least Rs 4,200 (US$84) to below poverty line (BPL) households in the village. The Indian Ministry of Rural Development classifies households as BPL using characteristics such as land holdings, house type, consumer durables, and literacy [20]. BPL households were identified in this study by their ration card color (a document used to access public food and grain distribution system). While the TSC provided subsidy of Rs 2,200 (US$44) to BPL households, *Nirmal Vatika* provided additional at least Rs 2,000 (US$40) to BPL and non-BPL households both to support IHL construction. These costs were determined by the government to be adequate to construct an offset two-pit latrine with water sealed squat plate and a brick walled room (which will be a JMP defined improved sanitation facility), and this type of latrine was actively promoted in the study districts.

Beginning in 2006, the WSP India office supported the TSC program under the TSSM project in ten districts in Madhya Pradesh. The WSP worked with local authorities to create an enabling environment for the TSC activities, to develop local implementation capacities at the district level, and to support the use of monitoring systems to assess progress towards the TSC goals. WSP promoted and provided capacity building support to implement community-led total sanitation (CLTS) based behavior change methods [21]. The CLTS methodology involves a series of community “triggering” exercises, led by an external facilitator after building rapport with the community in the pre-triggering phase, which highlight the magnitude of the practice of open defecation, elicit shame and disgust, and mobilize community action to end open defecation [21]. These triggering activities are followed by community follow-up actions that are supported by facilitators. Although the intervention used CLTS based tools for behavior change, it cannot be considered as a classical CLTS intervention. CLTS principles require that no hardware subsidies be provided to individual households and specific latrine models not be prescribed [21], whereas the intervention provided hardware subsidies to individual households to build offset pit latrine designs approved under the *Nirmal Vatika* program. Provision of hardware subsidy as a post-construction incentive was advocated by the WSP, but the mechanisms of the convergence of *Nirmal Vatika* and the TSC essentially meant that the subsidies were released before and during but rarely after IHL construction.

The TSC program in the study areas was implemented by the village government (*Gram Panchayat*) with support from district and block administration personnel or consultants. The study investigators and staff were not involved in program implementation.

### Outcome Definition and Measurement

The study measured outcomes using a combination of structured questionnaires and observations, sampling and testing of drinking water, child anthropometry and specimen (stool and blood) testing. GfK Mode Pvt Ltd. was contracted to conduct the fieldwork. Training and all field activities were overseen by the study investigators (SRP, ALS). The baseline survey was conducted between 25 May and 18 July, 2009, and the follow-up survey was conducted between 23 February and 25 April, 2011. Questionnaires used in the follow-up survey were the same as those used in the baseline survey with some additional questions to measure program exposure and outcomes. The household questionnaire collected information about household socioeconomics, demographics, exposure to the TSC activities, water and sanitation infrastructure, sanitation- and hygiene-related behaviors, and health/diseases. Interviewers conducted standardized spot-check observations of dwelling sanitation and hygiene facilities. Defecation behavior was reported by adults during private, in-home interviews. Main outcomes were defined as follows.

#### Toilets, open defecation, hygienic conditions

We classified household sanitation facilities using questions and definitions proposed by the JMP [2]. JMP-defined improved sanitation includes flush/pour flush toilet connected to piped sewer, to septic tank or to offset pit, ventilated improved pit latrine, on-pit latrine with slab and composting toilet that can hygienically separate human excreta from human contact. However, it is possible that the households build rudimentary latrines that are not included in the JMP definition of improved sanitation. For example, in addition to no facility or open defecation, the JMP defined unimproved sanitation facilities include flush toilets disposing waste elsewhere, pit latrine without a slab (open hole), bucket latrine, and hanging latrine. We also report availability of all types of IHLs whether improved or unimproved to assess whether the households moved up the sanitation ladder from no facility to some type of latrine even if unimproved. To assess defecation behavior for men, women, and children (<5 years), interviewers asked households separately for each group whether they openly defecate daily/always, occasionally/seasonally, or never. Interviewers also asked about child feces disposal using the standard JMP question [2]; disposal in a toilet, a confined pit, or buried was classified as hygienic. Field staff also observed whether the IHLs (of any type if present) were being used on the basis of worn path, closable door, odor, anal cleaning material, and water to flush. Field staff also recorded any observed human or animal feces in the household living area.

#### Caregiver reported illness

The study's primary outcome was diarrhea and HCGI among children <5 years old. We defined diarrhea as ≥3 loose or watery stools in 24 hours, or a single stool with blood/mucus [22] with a 7-day recall period [23] using a previously published instrument [24]. HCGI—a more inclusive measure of enteric infection—was defined as any of the following four conditions: (1) diarrhea; (2) vomiting; (3) soft or watery stool and abdominal cramps occurring together on any day; or (4) nausea and abdominal cramps occurring together on any day [25]–[28]. We measured respiratory symptoms (constant cough, pulmonary congestion, difficulty breathing, breaths per minute) and defined acute lower respiratory illness (ALRI) as constant cough or difficulty breathing and a raised respiratory rate [29]. We also measured bruising/abrasions and itchy skin/scalp to serve as negative control outcomes [30] to check for differential reporting bias in this unblinded trial [31],[32].

#### Anthropometry

We measured children <24 months at enrollment for height, weight, and mid-upper arm circumference (MUAC) using a standardized anthropometry protocol [33],[34]. Pairs of trained anthropometrists measured child length/height to the nearest 0.1 cm using a portable stadiometer (manufacturer: Seca); children <24 months were measured in the recumbent (lying) position and older children (at follow-up) were measured standing. Weight was measured to the nearest 0.1 kg using an electronic scale (manufacturer: Tanita); children unable to stand were weighed in their caregiver's arms and the caregiver's weight measured separately. MUAC was measured to the nearest 0.1 cm using a pediatric measuring tape. All measurements were collected in duplicate and we used the average of the two measurements in the analysis. We excluded observations if the two measurements differed by >10% (*n* = 21 [0.48%] for height, *n* = 85 [1.93%] for weight, *n* = 23 [0.52%] for MUAC). We converted the anthropometric measurements into Z-scores using the WHO's 2006 growth standards and the WHO publicly available Stata algorithm [35].

#### Anemia

If the caregiver provided informed consent, trained field staff conducted an in-field test for anemia for children between the ages of 6 and 60 months using HemoCue (HemoCue Ltd). We classified children as severely anemic if their hemoglobin concentration was <7.0 g/dl, moderately anemic if their hemoglobin concentration was 7.0–9.9 g/dl, and mildly anemic if their hemoglobin concentration was 10.0–11.9 g/dl [36]. Parents of children who were severely anemic were advised to visit the nearest health facility for medical attention.

#### Water quality

We collected 100 ml stored drinking water samples from a random sample of 404 households in the intervention and 403 households in the control groups, and also collected paired samples from the water source from which the households collected their drinking water (511 source samples). The water samples were collected in sterile containers, labeled, and individually packed in a sterile plastic zip-lock cover provided by the laboratory. The sample collectors were provided with sterile gloves and trained to avoid cross-contamination of water and containers. Water samples were stored and transported in ice boxes and tested for *Escherichia coli* using membrane filtration (100 ml volume filtered) within 36 hours of collection at Envirocare Laboratories Pvt Ltd, Mumbai. The laboratory used HiCrome Agar (M1466) by HiMedia. Each incubation batch included positive and negative control plates. Positive colonies of *E. coli* were further confirmed with Triple Sugar Iron (TSI) agar test and group of Indole, Methyl red, Voges-Proskauer, and Citrate tests (IMViC). Samples below the lower limit of detection were imputed at 0.5 colony forming units (CFU) per 100 ml (half the limit of detection [37]), and samples beyond the upper limit of detection were imputed at the limit of detection (200 CFU/100 ml).

#### Child stool parasitology

At the follow-up survey, we selected a random subsample of 1,150 households from 3,039 households and collected a stool specimen from the oldest child between 21 and <60 months of age. All stool samples were preserved in 10% formalin and analyzed at the National Institute for Cholera and Enteric Diseases in Kolkata. Lab technicians tested the samples for soil transmitted helminthes (*Ascaris lumbricoides*, *Trichuris trichiura*, *Ancylostoma duodenale*, and *Necator americanus*) and tapeworm helminthes (*Hymenolepis nana, Taenia sp., Diphyllobothrium latum*) using the Kato-Katz technique [38].

A separate aliquot was analyzed to test for protozoan infections (*Giardia lamblia, Cryptosporidium sp., Entamoeba histolytica*) using a commercially available ELISA kit (TechLab) [39],[40]. All specimens were tested with a combination of microscopy, ELISA, and PCR to achieve high levels of sensitivity and specificity. If a child tested positive for one of the protozoan infections using either microscopy or ELISA, the result was confirmed using isolated DNA from the ELISA positive samples followed by PCR-restriction fragment length polymorphism (RFLP) methods for genotyping local isolates of giardia (β-giardin) [32], Cryptosporidium (18s rRNA) [33], and *E. histolytica* (SSU rRNA) [34]. If a sample tested positive by microscopy or ELISA but was not confirmed by molecular methods then the sample was classified as negative.

### Sample Size

The study was originally designed to have 80% power to detect a 4.5 percentage point reduction in diarrhea prevalence among children <5 years old assuming 15% prevalence in the control group (or a 30% relative reduction) with a two-sided alpha of 5%, and an intra-class correlation of 0.105 [41]. These assumptions led to a design with 40 clusters (villages) per arm and 25 households with children <24 months per cluster. After the commencement of the study but without knowledge of any study outcomes, we decided to additionally power the trial to detect differences between groups in height-for-age Z-scores on the basis of a hypothesis published on the possible effects of improved hygiene and sanitation on child growth [14]. We reviewed measures of variability and within-cluster correlation of height-for-age Z-scores (SD = 2.09, intra-class correlation = 0.17), and chose to increase the within-cluster sample sizes from 25 to 38 households to ensure the study had 80% power to detect differences of +0.2 Z in height-for-age.

### Randomization

The village-level randomization was stratified at the administrative Block level because the TSC implementation was coordinated at the Block level and we wanted to ensure that the treatment arms were evenly allocated between districts and geographically stratified within districts. The randomization took place in a public lottery led by study investigators. The Block TSC coordinators or their representatives picked the lottery ticket that assigned villages to treatment groups. Overall, we allocated a total of 20 villages in each district to the intervention and 20 to control (40 villages per arm). The program implementers and researchers were not blinded to the group assignment. Field interviewers were not informed of group assignment, but it was possible for them to identify intervention villages during interviews of Block officers or the village secretary.

### Statistical Methods

We checked the baseline balance in the observable characteristics of the randomized groups. Due to highly comparable groups at baseline and the large increase in our within-cluster sample between baseline and follow-up, our analysis focused on group comparisons post-intervention (using follow-up measures only). To evaluate any differential effect of attrition (loss to follow-up) between baseline and follow-up, we compared baseline characteristics of those present at follow-up with those lost to follow-up. We also compared the balance of baseline characteristics across treatment groups for individuals who were present at both baseline and follow-up to determine whether attrition was differential by treatment group.

Our parameter of interest for all outcomes was the mean difference between randomized groups. We conducted the analysis using households and individuals as they were randomized (intention to treat [ITT]). We estimated differences between groups using the following linear regression model:
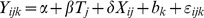
(1)Where, *Y_ijk_* is the outcome for individual *i* in village *j* and Block *k*; *T_j_* is the intervention indicator (1 for intervention, 0 for control); *X_ij_* are individual, household, and village level characteristics used in adjusted analyses; *b_k_* are indicator variables for Blocks since randomization was stratified at the Block level; and *ε_ijk_* is the error term. The parameter *β* estimates the ITT difference between the randomized groups. In the adjusted analyses, we included the following covariates to improve precision: whether the household head had attended school; whether the government categorized the household as Scheduled Caste or Tribe; child age; and child sex. Additionally, the adjusted models included three baseline characteristics found to be slightly imbalanced between groups despite randomization. These included: percentage of households in the village that used improved water sources; percentage of households in the village that were observed to have soap and water at the hand-washing place used after defecation; and mean height-for-age Z-score of children in the village. To further assess differential impacts of the program by important population subgroups, we re-estimated the effect of the intervention for households with and without IHL (any type) at baseline, and households below the official poverty line and the other households.

Since we would expect behaviors and child health outcomes to be correlated within villages, all estimates used Huber-White robust standard errors for the parameter β clustered at the village level [42] and reported *p*-values for the two sided t-test. Following guidance from Schulz and Grimes [43], we did not adjust *p*-values or confidence intervals for multiple comparisons because many of the outcomes were highly correlated with one another (for example, correlation between primary outcomes diarrhea and HCGI = 0.78); nominal *p*-values should be interpreted with this in mind. All analyses were conducted using Stata v12 (Statacorp), and all primary analyses were independently replicated by two investigators (SRP, BFA) from untouched datasets to final estimates.

### Access to Protocol and Data

The study protocol, questionnaires, and access to data collected in the study are available upon registration at http://microdata.worldbank.org/.

## Results

### Enrolment, Baseline Balance, and Attrition

Figure 2 depicts the study participants flow. The baseline survey enrolled a sample of 3,390 children <5 years from 1,954 households from 80 villages. In the follow-up survey the sample size was increased to 5,209 children <5 years from 3,039 households. As reported in Table 2, baseline covariates in intervention and control groups were well balanced with four exceptions. First, 89% of the households in the intervention group had access to improved water sources—tap/piped water, tube well and protected dug wells—compared to 80% of households in the control group. In contrast, a larger proportion of control households (54%) were observed to have soap and water at hand-washing locations used after defecation than in intervention households (44%). On average, more children were found to be anemic in the control group (93%) than in the intervention group (88%). Finally, average height-for-age Z-scores were also slightly imbalanced (−1.38 intervention versus −1.81 control).

**Table 2:** Baseline characteristics by randomized intervention groups, 2009.

Characteristics	Intervention (I)		Control (C)	
	N^a^	Mean or Percent	N^a^	Mean or Percent
**Household characteristics**				
Age in months for children <5 years^b^	1,683	21.89	1,707	22.12
Age of HH head in years	976	45.34	978	43.18
Whether HH head went to school	954	49.90%	952	52.73%
Government category of HH as BPL	976	34.53%	978	38.96%
Government category of HH as schedule caste/tribe	935	69.73%	905	71.38%
*Pucca* (better quality) HH construction	976	57.07%	978	60.43%
Monthly HH income (Rupees)	976	11,293	978	11,022
**WASH infrastructure and behaviors**				
HH access to improved water source	976	89.24%	978	79.65%
Reported drinking water treatment at home	976	68.34%	978	66.26%
Interviewer observed soap and water at hand-washing place used post defecation	969	44.48%	972	54.22%
PCG reports hand washing w/soap after fecal contact in last 24 hours	978	61.76%	985	64.16%
**Child nutrition**				
Child ever breastfed^c^	1,026	99.03%	1,037	98.55%
Child still breastfeeding^c^	1,013	91.21%	1,021	89.52%
Iron pills, syrup given^c^	1,019	7.36%	1,033	5.91%
Drugs for intestinal worms given in past 6 months^c^	1,025	19.12%	1,033	15.97%
Did receive VitA dose last 6 months^c^	1,013	37.41%	1,032	36.14%
**Sanitation**				
Reported main sanitation facility is JMP defined improved sanitation facility	975	13.64%	978	12.37%
Reported main sanitation facility is any type of IHL/is not open defecation	975	18.36%	978	20.96%
Reported correct disposal of child feces	976	15.98%	978	13.39%
Interviewer did not observe feces in living area around HH	973	41.11%	976	38.11%
**Water microbiology**				
HH drinking water is contaminated with *E. coli*	172	95.93%	174	97.70%
**Health status**				
Diarrhea 7-day prevalence^b^	1,683	13.19%	1,707	12.13%
HCGI 7-day prevalence^b^	1,683	15.27%	1,707	15.06%
ALRI 7-day prevalence^b^	1,683	11.47%	1,707	10.13%
Weight-for-age Z-score^c^	957	−2.20	943	−2.18
Length/height-for-age Z-score^c^	932	−1.38	933	−1.81
Arm circumference-for-age Z-score^c^	921	−1.31	895	−1.33
Weight-for-height Z-score^c^	895	−1.68	879	−1.43
Anemic: Hb<110 g/l^c^	293	88.05%	329	92.71%

a *N* is the base number of observations (the denominator) for the reported percentages or the sample size used to estimate the reported means. *N* is the number of households except for the variables measured at the child level (as indicated by **^b^** and **^c^**) where *N* is the number of children. *N* varies across different variables because of measurements in only a subset of the sample by design, non-response/refusal, and the loss due to measurement errors.

b For children less than 60 months of age.

c For children less than 24 months of age.

ALRI, acute lower respiratory illness; CFU, colony forming units; Hb, Hemoglobin; HH, household; PCG, primary care giver; VitA, vitamin A; WASH, water, sanitation and hygiene.

Attrition was not differential by randomized group on the basis of observable characteristics (see Table S1). Of the 1,954 households enrolled at the baseline, 1,655 were located at the 21-month follow-up survey (15% attrition) without any significant difference between the intervention (16%) and the control (15%) groups. Characteristics remained balanced between intervention and control groups in remaining households.

### Compliance to Randomization

The study measured intervention implementation in multiple ways because of the complexity of the TSC program. These measures included: reported implementation by Block coordinators, expenditure of funds documented by official program records, and interviews with local village officials. Out of 40 intervention villages, staff collected administrative information on 39 villages from the TSC Block coordinators (government officers). The coordinators reported that 15/39 intervention villages received some CLTS activities, 33/39 villages applied for a NGP award prior to the follow-up survey. According to Block coordinators' records, 25/39 villages had 100% households with IHLs, 11/39 villages had 80%–99% households with IHLs, and three of 39 villages with 37%–68% households with IHLs. Block coordinators also reported that 21/39 villages received 100% of the funds allocated under the TSC program, 12/39 villages received between 50% and 99%, and six of 39 villages received <50% of their allocated funds. The latest disbursement of the TSC funds was given to 36/39 intervention villages at least 4 to 5 months before the follow-up survey, which would offer sufficient time for IHLs to be constructed and used for 3 or more months.

The study review meetings with Block coordinators also identified that some control villages were contaminated during the study period: TSC activities were initiated in eight control villages within a few months of baseline survey and possibly in two additional control villages a few months prior to the follow-up survey; official records were not available for control villages to ascertain this information objectively. As per the follow-up survey in these ten contaminated villages, the household level coverage of JMP defined improved sanitation facilities increased from 17.4% at baseline to 41.4% at the follow-up, which is similar to the program effect we observed in the intervention group. The household level coverage of JMP defined improved sanitation facilities in uncontaminated control villages increased from 10.7% to 16.2% in the same period. The study's long follow-up period (21 months) and the highly publicized and politicized nature of the TSC program may have contributed to this contamination.

Information from additional sources (village secretaries, school teachers, *Anganwadi* [pre-school] workers in the village, and the rapid assessment from random sample of households) confirmed that TSC activities translated into a higher recollection and knowledge of the TSC program in the intervention villages compared to the control villages. We also found that households in intervention villages were more aware of CLTS activities, had higher knowledge of TSC, and experienced more personal visits to convince them to build and use IHLs (Table 3).

**Table 3:** Effect of the intervention on program outputs, behavioral outcomes, and water quality, 2011.

Outputs and Outcomes	Control Group^a^		Intervention Group^a^		ITT Unadjusted^b^	ITT Adjusted^c^
	*N*	Mean	*N*	Mean	Difference [95% CI]^d^	Difference [95% CI]^d^
**Program exposure**						
HH received WASH message from mass media	1,511	0.272	1,523	0.295	0.023 [−0.033 to 0.080]	0.000 [−0.048 to 0.048]
HH received WASH message from personal visits	1,472	0.099	1,479	0.240	0.140 [0.097–0.183]***	0.127 [0.081–0.172]***
HH participated or is aware of CLTS activities	1,514	0.157	1,525	0.291	0.135 [0.083–0.186]***	0.140 [0.089–0.191]***
HH knew of TSC/NGP	1,514	0.211	1,525	0.273	0.062 [0.011–0.114]**	0.053 [0.004–0.103]**
**Drinking water supply and hand-washing Infrastructure**						
HH access to improved water source	1,514	0.949	1,525	0.970	0.021 [−0.001 to 0.043]*	0.007 [−0.014 to 0.027]
Interviewer observed soap and water at hand-washing place used post defecation	1,269	0.436	1,334	0.494	0.056 [−0.006 to 0.118]*	0.052 [−0.002 to 0.105]*
**IHL access and sanitation behaviors**						
HH with JMP defined improved sanitation facilities	1,512	0.226	1,522	0.414	0.188 [0.118–0.258]***	0.177 [0.107–0.246]***
HH with any type of IHL	1,514	0.242	1,525	0.441	0.198 [0.126–0.270]***	0.189 [0.116–0.263]***
Interviewer assessed that HH is using IHL (any type)	1,504	0.167	1,520	0.272	0.104 [0.047–0.161]***	0.093 [0.042–0.144]***
Reported daily OD by men	1,514	0.841	1,525	0.746	−0.095 [−0.152 to −0.039]***	−0.087 [−0.135 to −0.038]***
Reported daily OD by women	1,514	0.835	1,525	0.732	−0.102 [−0.159 to −0.045]***	−0.091 [−0.141 to −0.041]***
Reported daily OD by children	1,514	0.892	1,525	0.839	−0.053 [−0.095 to −0.011]**	−0.054 [−0.088 to −0.020]***
Reported correct child feces disposal	1,514	0.184	1,525	0.271	0.087 [0.045–0.129]***	0.075 [0.036–0.113]***
Interviewer did not observe human/animal feces in HH living area	1,500	0.398	1,512	0.404	0.006 [−0.045 to 0.057]	0.019 [−0.026 to 0.065]
**Drinking water quality**						
*E. coli* present in household drinking water	403	0.821	404	0.767	−0.055 [−0.111 to 0.000]*	−0.032 [−0.101 to 0.036]
*E. coli* present in the source from where household collected drinking water	280	0.743	231	0.701	−0.115 [−0.269, 0.040]	−0.016 [−0.180, 0.149]

a The number of observations used to estimate means of the intervention and the control groups is the same as the number of observations used in ITT-unadjusted analysis.

b Explanatory variables in the unadjusted model include the treatment assignment and indicator variables for Blocks. Therefore, the ITT effects for outcomes may not be exactly the difference between the listed mean in intervention and control groups in previous columns.

c Because of missing adjustment variables data, the observations used in adjusted analysis are fewer than those used in unadjusted analysis. The number of observations used is seven to 113 less than that in unadjusted analysis.

d Following the commonplace norms, statistical significance is indicated as:

*** significant at α = 0.01;

** significant at α = 0.05;

* significant at α = 0.10.

Please note that *p*-values are not adjusted for multiple comparisons following guidance from Schulz and Grimes [43].

CFU, colony forming units; HH, household; OD, open defecation; TSC/NGP, Total Sanitation Campaign/*Nirmal Gram Puraskar*; WASH, water, sanitation and hygiene.

### IHL Coverage and Sanitation-Related Behaviors

Table 3 reports the intervention's effect on IHL availability (JMP defined improved sanitation facilities and any type of IHLs) and open defecation behaviors by household members. The intervention increased the coverage of JMP defined improved sanitation facility by average 19 percentage points (95% CI 12%–26%; *p*-value<0.001) in intervention villages compared to control villages (41.4% intervention versus 22.6% control). The intervention increased the coverage of any type of IHL facility by 20 percentage points (95% CI 13%–27%; *p*-value<0.001) in intervention villages compared to control villages (44.1% intervention versus 24.2% control). These results indicate that available IHLs were predominantly JMP defined improved sanitation facilities and very few rudimentary latrines or latrines defined as unimproved by the JMP were built. These results are consistent with the TSC design that promoted latrine models that can safely contain the feces.

Although on average fewer households in intervention villages were likely to report daily open defecation compared to control villages for adult men (75% intervention versus 84% control; mean difference: 9.5%; *p*-value = 0.001), adult women (73% intervention versus 83% control; mean difference: 10%; *p*-value<0.001), and children <5 years (84% intervention versus 89% control; mean difference: 5%; *p*-value = 0.014), these reductions in reported open defecation behaviors were smaller than the gains in IHL availability. Amongst the 630 households in intervention villages that had JMP defined improved sanitation facilities at follow-up, 41% reported that adult men or women still practiced daily open defecation; this same figure was 28% among the 339 control village households at follow-up (not reported in results table). A follow-up debriefing question to households who had IHL identified that the main reasons for daily open defecation in spite of having IHL were culture, habit, or preference for defecating in open followed by inadequate water availability.

### Drinking Water Quality

In control villages, 82% (331/403) of household drinking water samples tested positive for *E. coli* compared to 77% (310/404) of samples in intervention villages (mean difference: 5.5%; *p*-value = 0.050) (Table 3). Table S2 lists the distribution of positive household samples by different *E. coli* contamination level categories.

Of 511 water source samples tested, 74% (208/280) of the sources in control villages and 70% (162/231) in intervention villages tested positive for *E. coli* but the difference was not statistically significant (*p*-value = 0.143).

### Caregiver Reported Illness

Diarrhea prevalence did not differ between groups (7.4% intervention versus 7.7% control; *p*-value = 0.687) (Table 4). HCGI prevalence also did not differ between groups (11.5% intervention versus 12.0% control; *p*-value = 0.692) (Table 4). We observed no significant differences between groups in negative control caregiver-reported outcomes including bruising/abrasions (1.4% intervention versus 1.3% control) and itchy skin/scalp (2.5% intervention versus 2.2% control) suggesting that differential outcome reporting bias for diarrhea and HCGI was unlikely.

**Table 4:** Effect of the intervention on health outcomes, 2011.

Health Outcomes	Control Group^a^		Intervention Group^a^		ITT Unadjusted^b^	ITT Adjusted^c^
	*N*	Mean	*N*	Mean	Difference [95% CI]^d^	Difference [95% CI]^d^
**Caregiver reported illness in the last 7 days** e						
Diarrhea	2,609	0.077	2,600	0.074	−0.003 [−0.019 to 0.013]	−0.002 [−0.019 to 0.015]
HCGI	2,609	0.120	2,600	0.115	−0.004 [−0.026 to 0.017]	−0.002 [−0.024 to 0.020]
Acute lower respiratory illness	2,609	0.128	2,600	0.163	0.038 [0.003–0.073]**	0.049 [0.009–0.089]**
**Enteric parasite infections** f						
Any protozoan present	569	0.257	581	0.217	−0.040 [−0.089 to 0.008]	−0.027 [−0.082 to 0.029]
*Entamoeba histolytica* present	569	0.025	581	0.033	0.008 [−0.009 to 0.024]	0.009 [−0.009 to 0.028]
*Giardia lamblia* present	569	0.232	581	0.184	−0.048 [−0.096 to −0.001]**	−0.036 [−0.088 to 0.015]
Any helminth present	569	0.056	581	0.059	0.001 [−0.021 to 0.023]	−0.005 [−0.028 to 0.018]
*Ascaris lumbricoides* present	569	0.044	581	0.043	−0.002 [−0.021 to 0.017]	−0.011 [−0.031 to 0.010]
Any enteric parasite present	569	0.309	581	0.270	−0.040 [−0.087 to 0.006]*	−0.032 [−0.083 to 0.020]
**Anemia and anthropometry** e						
Anemic: Hb<110 g/l	1,922	0.508	1,919	0.562	0.050 [−0.011 to 0.110]	0.033 [−0.030 to 0.096]
Child weight (to 0.1 kg)	2,161	10.277	2,154	10.069	−0.229 [−0.492 to 0.033]*	−0.130 [−0.345 to 0.085]
Child height (to 0.1 cm)	2,185	82.312	2,175	81.682	−0.678 [−1.362 to 0.006]*	−0.242 [−0.789 to 0.304]
Child arm circumference (to 0.1 cm)	2,191	13.805	2,197	13.783	−0.004 [−0.145 to 0.138]	−0.022 [−0.167 to 0.123]
Weight-for-age Z-score	2,161	−1.833	2,154	−1.921	−0.095 [−0.253 to 0.063]	−0.094 [−0.246 to 0.058]
Length/height-for-age Z-score	2,185	−2.155	2,175	−2.189	−0.034 [−0.195 to 0.127]	−0.040 [−0.223 to 0.144]
MUAC-for-age Z-score	2,191	−1.337	2,197	−1.337	0.020 [−0.115 to 0.155]	−0.022 [−0.151 to 0.108]
Weight-for-height Z-score	2,054	−0.834	2,054	−0.847	−0.018 [−0.195 to 0.160]	0.029 [−0.142 to 0.199]
BMI Z-score	2,052	−0.604	2,052	−0.664	−0.062 [−0.241 to 0.117]	−0.019 [−0.191 to 0.153]

a The number of observations used to estimate means of the intervention and the control groups is the same as the number of observations used in ITT-unadjusted analysis.

b Explanatory variables in the unadjusted model include the treatment assignment and indicator variables for Blocks. Therefore, the ITT effects for outcomes may not be exactly the difference between the listed mean in intervention and control groups in previous columns.

c Because of missing adjustment variables data, the observations used in adjusted analysis are fewer than those used in unadjusted analysis.

d Following the commonplace norms, statistical significance is indicated as:

**significant at α = 0.05;

*significant at α = 0.10.

Please note that *p*-values are not adjusted for multiple comparisons following guidance from Schulz and Grimes [43].

e For children less than 60 months of age.

f For children less than 60 months of age. The eldest child less than 60 months of age selected from a household.

BMI, body mass index; Hb, hemoglobin; HH, household; OD, open defecation.

### Enteric Parasite Infections

In the subsample of 1,150 children with stool collection, 5.7% (66/1150) had helminth infections and the majority (50/66) were *Ascaris* infections. All remaining infections were tapeworms; no children were infected with *T. trichiura* or hookworm. We observed no difference in helminth prevalence between intervention and control groups. *Giardia* infection was common, and consistent with slightly improved water quality in the intervention group, we found lower *Giardia* prevalence among children in intervention villages (18%) compared to children in control villages (23%) (mean difference: 4.8%; *p*-value = 0.047). We detected no *Cryptosporidium* infections in the study children, and a low prevalence of *E. histolytica* (33 out of 1,150; 2.9%).

### Anemia and Anthropometry

Anemia was prevalent in the study children (54%) and children were small according to international growth standards (Table 4). However, we found no differences between the randomized groups in anemia prevalence or growth outcomes.

### Subgroup Results

Table 5 presents the results of subgroup analyses of the effect of the intervention on households with or without any type of IHL at baseline and BPL or non-BPL households. As expected, the program had the largest improvements on JMP defined improved sanitation facilities, IHL use as assessed by enumerators, and reduced reported open defecation by household members in households that did not have IHL (any type) at baseline and in BPL households. This finding is consistent with the TSC design that targeted households without IHLs and offered larger IHL construction subsidies for BPL households. Among BPL households, the intervention increased JMP defined improved sanitation facilities coverage by 30 percentage points (48% intervention versus 18% control; *p*-value<0.001) and it reduced open defecation among women by 17 percentage points (73% intervention versus 90% control; *p*-value<0.001). Despite larger improvements in these intermediate outcomes among BPL households or households without IHL at baseline, we did not observe consistent improvement in health outcomes in these subgroups (Table 5).

**Table 5:** Differential effect of the intervention by population subgroups, 2011.

Characteristics	Control Group^a^		Intervention Group^a^		ITT Unadjusted^b^	ITT Adjusted^c^
	*N*	Mean	*N*	Mean	Difference [95% CI]^d^	Difference [95% CI]^d^
**HH with JMP defined improved sanitation facilities**						
All HH	1,512	0.224	1,522	0.414	0.189 [0.119–0.259]***	0.178 [0.108–0.247]***
HH with IHL (any type) at baseline	190	0.979	212	0.967	−0.018 [−0.056 to 0.020]	0.001 [−0.027 to 0.029]
HH with no IHL (any type) at baseline	1,319	0.114	1,297	0.318	0.202 [0.139–0.264]***	0.209 [0.142–0.277]***
BPL HH	551	0.181	452	0.476	0.307 [0.227–0.388]***	0.320 [0.234–0.406]***
Non-BPL HH	961	0.249	1,070	0.388	0.135 [0.059–0.210]***	0.108 [0.027–0.189]***
**Reported daily OD by women**						
All HH	1,514	0.835	1,525	0.732	−0.102 [−0.159 to −0.045]***	−0.091 [−0.141 to −0.041]***
HH with IHL (any type) at baseline	191	0.105	214	0.103	0.000 [−0.078 to 0.077]	0.005 [−0.070 to 0.080]
HH with no IHL (any type) at baseline	1,320	0.941	1,297	0.837	−0.101 [−0.140 to −0.062]***	−0.097 [−0.140 to −0.054]***
BPL HH	551	0.902	453	0.733	−0.178 [−0.241 to −0.115]***	−0.169 [−0.233 to −0.105]***
Non-BPL HH	963	0.796	1,072	0.732	−0.061 [−0.129 to 0.006]*	−0.029 [−0.097 to 0.040]
***E. coli*** ** present in household drinking water**						
All HH	403	0.821	404	0.767	−0.055 [−0.111 to 0.000]*	−0.032 [−0.101 to 0.036]
HH with IHL (any type) at baseline	54	0.796	60	0.817	0.004 [−0.183 to 0.192]	−0.003 [−0.137 to 0.131]
HH with no IHL (any type) at baseline	347	0.827	340	0.765	−0.064 [−0.121 to −0.006]**	−0.055 [−0.135 to 0.026]
BPL HH	147	0.803	111	0.739	−0.069 [−0.169 to 0.031]	−0.076 [−0.198 to 0.047]
Non-BPL HH	256	0.832	293	0.778	−0.054 [−0.125 to 0.017]	−0.042 [−0.128 to 0.043]
**Diarrhea in the past 7 days** e						
All HH	2,609	0.077	2,600	0.074	−0.003 [−0.019 to 0.013]	−0.002 [−0.019 to 0.015]
HH with IHL (any type) at baseline	302	0.063	343	0.035	−0.034 [−0.072 to 0.003]*	−0.037 [−0.083 to 0.010]
HH with no IHL (any type) at baseline	2,302	0.079	2,231	0.080	0.001 [−0.016 to 0.018]	0.003 [−0.015 to 0.021]
BPL HH	949	0.085	783	0.078	−0.005 [−0.031 to 0.021]	0.004 [−0.022 to 0.029]
Non-BPL HH	1,660	0.072	1,817	0.073	0.000 [−0.019 to 0.019]	−0.001 [−0.023 to 0.021]
***Ascaris lumbricoides*** ** infection** f						
All HH	569	0.044	581	0.043	−0.002 [−0.021 to 0.017]	−0.011 [−0.031 to 0.010]
HH with IHL (any type) at baseline	82	0.037	92	0.043	−0.004 [−0.051 to 0.043]	−0.005 [−0.087 to 0.078]
HH with no IHL (any type) at baseline	487	0.045	482	0.041	−0.004 [−0.025 to 0.017]	−0.013 [−0.033 to 0.006]
BPL HH	221	0.045	160	0.044	0.008 [−0.030 to 0.046]	0.023 [−0.026 to 0.072]
Non-BPL HH	348	0.043	421	0.043	−0.001 [−0.027 to 0.026]	−0.022 [−0.046 to 0.001]*
***Giardia lamblia*** ** infection** f						
All HH	569	0.232	581	0.184	−0.048 [−0.096 to −0.001]**	−0.036 [−0.088 to 0.015]
HH with IHL (any type) at baseline	82	0.232	92	0.185	−0.115 [−0.221 to −0.008]**	−0.060 [−0.206 to 0.086]
HH with no IHL (any type) at baseline	487	0.232	482	0.185	−0.041 [−0.094 to 0.011]	−0.036 [−0.094 to 0.023]
BPL HH	221	0.226	160	0.144	−0.073 [−0.141 to −0.005]**	−0.059 [−0.139 to 0.020]
Non-BPL HH	348	0.236	421	0.200	−0.041 [−0.098 to 0.016]	−0.027 [−0.088 to 0.035]

a The number of observations used to estimate means of the intervention and the control groups is the same as the number of observations used in ITT-unadjusted analysis.

b Explanatory variables in the unadjusted model include the treatment assignment and indicator variables for Blocks. Therefore, the ITT effects for outcomes may not be exactly the difference between the listed mean in intervention and control groups in previous columns.

c Because of missing adjustment variables data, the observations used in adjusted analysis are fewer than those used in unadjusted analysis.

d Following the commonplace norms, statistical significance is indicated as:

***significant at α = 0.01;

**significant at α = 0.05;

*significant at α = 0.10.

Please note that *p*-values are not adjusted for multiple comparisons following guidance from Schulz and Grimes [43].

e For children less than 60 months of age.

f For children less than 60 months of age. The eldest child less than 60 months of age selected from a household.

BPL, based on verification of household's food ration card; HH, household; non-BPL, households who do not have/show BPL ration card; OD, open defecation.

## Discussion

The TSC program, implemented with support of the WSP in Dhar and Khargone districts, increased household level coverage of JMP-defined improved sanitation facilities by a modest 19 percentage points in intervention villages compared to control (41% intervention versus 22% control; *p*-value<0.001). However, the reductions in reported open defecation by adults were even more modest: falling 9 to 10 percentage points (among men: 75% intervention versus 84% control; *p*-value = 0.001; among women: 73% intervention versus 83% control; *p*-value<0.001), while reports of correct child feces disposal increased because of intervention by 9 percentage points (27% intervention versus 18% control; *p*-value<0.001). The availability of IHL and the reductions in open defecation were higher in the BPL household or households without any IHL at the time of baseline but we did not find consistent improvements in the multiple health outcomes in these subgroups. The less than universal or very high levels of IHL coverage in the intervention villages combined with relatively small behavior changes are consistent with our finding of no improvements in child health outcomes including: diarrhea, enteric parasite infection, growth, and anemia.

The study's findings should be viewed as a measure of effectiveness for this specific implementation of India's TSC program in rural Madhya Pradesh. By the end of the study in the intervention group, coverage of JMP defined improved sanitation facilities in a village ranged between 5% and 79% households and percentage of households in a village reporting daily open defecation by adult men ranged between 32% and 97% and that by adult women ranged between 34% and 97%. It is unknown whether enteric pathogen risk is linearly or non-linearly related to the level of improved sanitation in a community, and the intervention did not achieve the goal of universal availability of IHLs or universal elimination of open defecation during the study period. Therefore, our findings cannot speculate the child health outcomes for universal or higher levels of IHL availability or larger open defecation reductions that may be feasible under different contexts, program designs, or implementation efficacy. Additional, forthcoming cluster randomized sanitation intervention trials [44],[45] may generate such evidence if they can achieve adequately high latrine coverage and proportional reductions in open defecation.

This study presents a cautionary tale of how difficult it can be to achieve universal IHL coverage or elimination of open defecation for scaled up rural sanitation programs. The study documented clear evidence of more social mobilization, exposure to behavior change activities, and IHL construction in intervention villages compared to control villages. However, these intermediate outputs of the TSC could not translate into high enough levels of IHL availability and reductions in open defecation practice to deliver the health impacts. This evaluation was a part of a broader six-country effort to also study large-scale sanitation promotion programs in rural Indonesia and Tanzania, as well as large-scale hand-washing promotion programs in Peru, Vietnam, and Senegal. While the Tanzania results are forthcoming, the Indonesia study found even smaller increases in availability of JMP defined improved sanitation facilities and reductions in open defecation following a large-scale sanitation campaign [46] that was similar in design to the classical CLTS approach [21]. A recent cross-sectional survey in Orissa found more optimistic results—72% IHL availability following the TSC [47]—but implementation was heterogeneous. Much less than universal levels of IHL coverage and use were reported in past evaluations of pilot programs and early implementations of India's TSC [48],[49].

Within the broader water-sanitation-hygiene sector, the difficulty of scaling up interventions that are efficacious when widely adopted and properly used across a community is not unique to rural sanitation. Evaluations of large-scale hand-washing promotion campaigns in Peru and Vietnam—part of the broader research effort that included the present trial—found almost no improvements in hand-washing behavior and thus no downstream impacts on child health [50],[51]. Furthermore, the interim evaluation of the national-level Sanitation Hygiene Education and Water supply in Bangladesh program found very small improvements in hygiene and sanitation outcomes, with no impacts on child health [52].

The present evidence from the sector suggests that with few exceptions [53] scaled up sanitation and hygiene programs in rural settings have had difficulty in delivering the health benefits measured in small efficacy studies. Typically, the well-controlled efficacy trials can result in high enough levels of sanitation and hygiene infrastructure and behaviors necessary to deliver the health benefits, but the same levels of infrastructure or behavior change are not guaranteed to accrue to large-scale programs. From a public health perspective, these findings call into question the likelihood of the TSC in its current form to improve child health. Still, the program may be valuable from the policy and development perspective for reasons beyond public health, such as the social benefits of sanitation (dignity, privacy, safety, and reduced burden of coping especially for women) accrued to households that have and use IHLs, and the obligation of the government to provide access to sanitation as a recently recognized human right by the United Nations General Assembly (Resolution number 64/292). As the next iteration of the TSC program—named *Nirmal Bharat Abhiyaan* (Clean India Campaign)—continues, research efforts that focus on how to significantly increase the access to and use of IHLs would be particularly valuable to guide future program refinement. High levels of IHL coverage and use should be demonstrated in pilot programs before these program refinements are taken to national scale.

### Limitations

Like other effectiveness studies that measure the impact of large-scale government programs, we faced the challenges typically not encountered in well-controlled efficacy trials such as imperfect compliance with treatment assignment and poor fidelity of intervention implementation. We found that by 21 months of follow-up, none of the intervention villages achieved the program goal of 100% households having and using IHLs that can safely confine feces; the average household level coverage of JMP defined improved sanitation facilities was 40% (range: 5%–79%). The reasons for the gap between the officials monitoring records of the TSC and the actual status are discussed elsewhere [54]. The Block coordinators also identified that at least eight and possibly ten control villages received the TSC program. ITT estimates of program impacts with imperfect compliance will underestimate the effect possible under perfect compliance.

Another challenge in trials where study investigators have limited control over the program implementation, is significant deviations in the actual implementation timeline compared to the timeline on which the evaluation study is based. While the planned follow-up period from the baseline was 18 months in this study, the actual follow-up measurement at 21 months was the latest possible point we could measure outcomes under the possibility of program expansion into control villages and contractual constraints with the evaluation funding. Although it was possible that impacts on diarrheal diseases could begin relatively soon after intervention, as documented in short-duration efficacy trials [55], we would expect impacts on enteric parasite infection, anemia, and growth to potentially accrue more slowly.

The limited length of follow-up could have also influenced our estimates of the program's effect on IHL availability and use. Longer follow-up could have led to potentially higher levels of IHL coverage or, conversely, lower levels of use (if IHLs are not maintained). Despite this limitation, our estimates of IHL coverage and reported use are broadly consistent with other independent measures following rural sanitation programs in India [47]–[49]. For example, Barnard and colleagues [47] found that 4 to 6 years after TSC implementation in Orissa that 53% of households with an IHL reported some individuals still practiced open defecation. In the present study, 41% of men and 38% of women from the intervention group who have JMP defined improved sanitation facilities reported practicing daily open defecation.

Self-reported outcomes can be subject to differential, biased reporting in unblinded trials [31],[32]. Therefore, in addition to self-reported illnesses, we included several objective child health measurements in this study (parasite infections, anemia, anthropometry). However, we did not include objective measures of sanitation behaviors (disposal of child feces, IHL use, and open defecation). To the extent that our measurements of reported outcomes were subject to courtesy bias, we may have over-estimated IHL use or under-estimated open defecation prevalence in the study population. Furthermore, if the bias was differential by treatment group, then we would expect the study to have over-estimated the improvements due to intervention because we would expect the intervention households to be more sensitized to the stigma of open defecation. Measures of IHL use could be improved in future sanitation studies through the use of passive sensors mounted in the latrine [56],[57].

### Generalizability

There is wide variation in TSC implementation within India, and it remains possible that the TSC program was more or less successful in other states [19]. We note, however, that very few Indian states had large growth in IHL availability between 2001 and 2011 when the TSC program was active across India. In Madhya Pradesh, the TSC program was combined with *Nirmal Vatika* that served to increase the IHL construction subsidies available to all eligible households. Additionally, the districts enrolled in this study received support from the WSP's TSSM project to build capacity for creating an enabling environment, record keeping and monitoring, and implementing CLTS-based behavior change approaches. Therefore, the behavior change approaches in the study districts were arguably more intensive than those in the rest of Madhya Pradesh. However, this study should be not viewed as an evaluation of the CLTS approach as advocated by its practitioners [21] because the intervention only used CLTS behavior change tools and did not follow the key principles of CLTS such as not providing hardware subsidy and not prescribing latrine models.

### Conclusions

This 80 village study in rural Madhya Pradesh represents the first published large-scale, randomized evaluation of India's TSC to measure and report outcomes at all stages of the causal chain (Figure 1). While the TSC program in rural Madhya Pradesh implemented with support from the WSP increased the household level availability of JMP defined sanitation facilities (+19%) and to a lesser extent reduced open defecation (−10%), these improvements were insufficient to improve child health outcomes (diarrhea, parasite infections, anemia, growth). Despite the limitations of the present study, including short follow-up and evidence for contamination in the control group, the results underscore the challenge of achieving adequately large levels of improvements in sanitation to deliver the expected health benefits within the scaled-up rural sanitation programs.

## Supporting Information

Table S1 **Analysis of balance in the baseline panel after attrition, Madhya Pradesh, 2009**
(DOC)

Table S2 **Distribution of** ***E. coli*** **contamination in household drinking water**
(DOCX)

Text S1 **CONSORT checklist**
(DOCX)

Text S2 **Follow-up study protocol**
(DOC)

## Abbreviations

BPL: below poverty line
CLTS: community led total sanitation
HCGI: highly credible gastrointestinal illness
IHL: individual household latrine
ITT: intention to treat
JMP: Joint Monitoring Programme
MUAC: mid-upper arm circumference
TSC: Total Sanitation Campaign
TSSM: Total Sanitation and Sanitation Marketing
WSP: Water and Sanitation Program (the World Bank)
