# Supplementary material for: The Effect of India's Total Sanitation Campaign on Defecation Behaviors and Child Health in Rural Madhya Pradesh: A Cluster Randomized Controlled Trial
Source: PLoS Med. 2014 Aug 26;11(8):e1001709. doi: 10.1371/journal.pmed.1001709 (PMC4144850; doi:10.1371/journal.pmed.1001709)
Supplement: Table S1 — Analysis of balance in the baseline panel after attrition, Madhya Pradesh, 2009 (DOC) [file pmed.1001709.s001.doc]

**Table S1. Analysis of Balance in the Baseline Panel after Attrition, Madhya Pradesh, 2009.**

| **Indicators** | **Attrition/Lost Households** | | | | | **Intact Households** | | | | |
| --- | --- | --- | --- | --- | --- | --- | --- | --- | --- | --- |
|  | **Intervention** | | **Control** | | **Mean Difference [95% CI]** | **Intervention** | | **Control** | | **Mean Difference [95% CI]** |
|  | **N** | **Mean** | **N** | **Mean** |  | **N** | **Mean** | **N** | **Mean** |  |
| **Household Characteristics** | | | | | | | | | | |
| Children <5 years age in months | 263 | 22.26 | 247 | 22.05 | 0.203 [-2.235, 2.642] | 1420 | 21.82 | 1460 | 22.13 | -0.314 [-1.254, 0.625] |
| Age of HH head in years | 154 | 41.26 | 145 | 42.74 | -1.478 [-5.766, 2.81] | 822 | 46.10 | 833 | 43.26 | 2.843 [0.506, 5.179] |
| Whether HH head went to school | 150 | 0.393 | 143 | 0.385 | 0.009 [-0.147, 0.165] | 804 | 0.519 | 809 | 0.553 | -0.034 [-0.126, 0.058] |
| Government category of HH as schedule caste/tribe | 150 | 0.853 | 130 | 0.769 | 0.084 [-0.061, 0.229] | 785 | 0.668 | 775 | 0.705 | -0.037 [-0.148, 0.074] |
| *Pucca* (high quality) HH construction | 154 | 0.481 | 145 | 0.600 | -0.119 [-0.264, 0.025] | 822 | 0.588 | 833 | 0.605 | -0.017 [-0.114, 0.079] |
| Monthly HH income | 154 | 4601 | 145 | 5790 | -1189 [-3869, 1491] | 822 | 12547 | 833 | 11933 | 614 [-1627, 2855] |
| **WASH Infrastructure and Behaviors** | | | | | | | | | | |
| HH access to improved water source | 154 | 0.864 | 145 | 0.862 | 0.002 [-0.095, 0.098] | 822 | 0.898 | 833 | 0.785 | 0.113 [0.019, 0.207] |
| Reported drinking water treatment at home | 154 | 0.597 | 145 | 0.641 | -0.044 [-0.16, 0.072] | 822 | 0.700 | 833 | 0.666 | 0.033 [-0.051, 0.117] |
| Interviewer observed soap and water at hand washing place used post defecation | 153 | 0.327 | 144 | 0.396 | -0.069 [-0.216, 0.078] | 816 | 0.467 | 828 | 0.568 | -0.101 [-0.226, 0.024] |
| PCG reports hand washing w/ soap after fecal contact in last 24 hours | 151 | 0.556 | 145 | 0.593 | -0.037 [-0.163, 0.089] | 827 | 0.629 | 840 | 0.650 | -0.021 [-0.108, 0.066] |
| **Child Nutrition** | | | | | | | | | | |
| Child ever breastfed**^&^** | 160 | 0.969 | 153 | 0.993 | -0.025 [-0.058, 0.008] | 866 | 0.994 | 884 | 0.984 | 0.010 [-0.001, 0.021] |
| Child still breastfeeding**^&^** | 153 | 0.922 | 152 | 0.868 | 0.053 [-0.026, 0.132] | 860 | 0.910 | 869 | 0.900 | 0.011 [-0.015, 0.036] |
| Iron pills, syrup given**^#^** | 158 | 0.089 | 153 | 0.065 | 0.023 [-0.041, 0.087] | 861 | 0.071 | 880 | 0.058 | 0.013 [-0.018, 0.044] |
| Drugs for intestinal worms given past 6**^#^** | 160 | 0.181 | 153 | 0.157 | 0.024 [-0.078, 0.126] | 865 | 0.193 | 880 | 0.160 | 0.033 [-0.015, 0.080] |
| Did receive VitA dose last 6 months**^#^** | 156 | 0.256 | 152 | 0.329 | -0.073 [-0.179, 0.034] | 857 | 0.312 | 877 | 0.315 | -0.003 [-0.063, 0.057] |
| **Program Outputs/Outcomes** | | | | | | | | | | |
| HH with improved sanitation (toilets) | 154 | 0.052 | 145 | 0.083 | -0.031 [-0.136, 0.074] | 821 | 0.152 | 833 | 0.130 | 0.023 [-0.047, 0.092] |
| Reported correct disposal of child feces | 154 | 0.097 | 145 | 0.117 | -0.020 [-0.104, 0.064] | 822 | 0.172 | 833 | 0.137 | 0.035 [-0.023, 0.093] |
| Interviewer did not observe feces in living area around HH | 152 | 0.408 | 144 | 0.340 | 0.068 [-0.043, 0.178] | 821 | 0.412 | 832 | 0.388 | 0.023 [-0.074, 0.121] |
| **Water Microbiology** | | | | | | | | | | |
| Log_10_ *E. coli* in household drinking water (CFU/100 ml) | 24 | 2.082 | 22 | 1.987 | 0.096 [-0.288, 0.480] | 148 | 1.999 | 152 | 2.073 | -0.074 [-0.254, 0.106] |
| HH drinking water is contaminated with *E. coli* | 24 | 0.958 | 22 | 0.955 | 0.004 [-0.114, 0.122] | 148 | 0.959 | 152 | 0.980 | -0.021 [-0.062, 0.020] |
| **Health Status** | | | | | | | | | | |
| Diarrhea 7-day prevalence**^#^** | 263 | 0.125 | 247 | 0.126 | 0.000 [-0.070, 0.070] | 1420 | 0.133 | 1460 | 0.121 | 0.013 [-0.028, 0.053] |
| ALRI 7-day prevalence**^#^** | 263 | 0.137 | 247 | 0.113 | 0.024 [-0.068, 0.115] | 1420 | 0.111 | 1460 | 0.099 | 0.011 [-0.049, 0.071] |
| Weight-for-age z-score | 141 | -2.410 | 131 | -2.141 | -0.269 [-0.579, 0.042] | 816 | -2.159 | 812 | -2.186 | 0.028 [-0.153, 0.209] |
| Length/height-for-age z-score**^#^** | 141 | -1.408 | 126 | -1.542 | 0.135 [-0.538, 0.807] | 791 | -1.378 | 807 | -1.848 | 0.470 [0.035, 0.905] |
| Arm circumference-for-age z-score**^#^** | 139 | -1.519 | 122 | -1.371 | -0.148 [-0.603, 0.307] | 782 | -1.273 | 773 | -1.324 | 0.051 [-0.188, 0.291] |
| Weight-for-height z-score**^#^** | 135 | -1.850 | 118 | -1.565 | -0.284 [-0.748, 0.179] | 760 | -1.651 | 761 | -1.411 | -0.240 [-0.561, 0.081] |
| Anemic: Hb < 110 g/l**^#^** | 43 | 0.884 | 36 | 0.944 | -0.061 [-0.222, 0.100] | 250 | 0.880 | 293 | 0.925 | -0.045 [-0.093, 0.004] |

**Abbreviations –** HH: Household; PCG: Primary Care Giver; VitA: Vitamin A; CFU: Colony Forming Units; E. Coli: Escherichia coliform; ALRI: Acute Lower Respiratory Illness; Hb: Hemoglobin

**&** For children less than 24 months of age

**#** For children less than 60 months of age
