# Supplementary material for: The Effect of India's Total Sanitation Campaign on Defecation Behaviors and Child Health in Rural Madhya Pradesh: A Cluster Randomized Controlled Trial
Source: PLoS Med. 2014 Aug 26;11(8):e1001709. doi: 10.1371/journal.pmed.1001709 (PMC4144850; doi:10.1371/journal.pmed.1001709)
Supplement: Table S2 — Distribution of E. coli contamination in household drinking water (DOCX) [file pmed.1001709.s002.docx]

**Table S2. Distribution of *E. coli* Contamination in Household Drinking Water**

| ***E. coli* Contamination Level** | **Control Group** | | **Intervention Group** | | **Total** | |
| --- | --- | --- | --- | --- | --- | --- |
|  | **N** | **Mean** | **N** | **Mean** | **N** | **Mean** |
| < 1 CFU / 100 mL | 72 | 17.87% | 94 | 23.27% | 166 | 20.57% |
| 1 to 99 CFU / 100 mL | 164 | 40.69% | 159 | 39.36% | 323 | 40.02% |
| 100-199 CFU / 100 mL | 46 | 11.41% | 31 | 7.67% | 77 | 9.54% |
| 200 or more CFU / 100 mL | 121 | 30.02% | 120 | 29.70% | 241 | 29.86% |
| *TOTAL* | *403* | *100%* | *404* | *100%* | *807* | *100%* |

***Abbreviations –*** CFU: Colony Forming Units; mL: milliliter; *E. coli*: Escherichia coli
