# Supplementary material for: The Effect of India's Total Sanitation Campaign on Defecation Behaviors and Child Health in Rural Madhya Pradesh: A Cluster Randomized Controlled Trial
Source: PLoS Med. 2014 Aug 26;11(8):e1001709. doi: 10.1371/journal.pmed.1001709 (PMC4144850; doi:10.1371/journal.pmed.1001709)
Supplement: Text S2 — Follow-up study protocol (DOC) [file pmed.1001709.s004.doc]

**SCALING UP SANITATION PROJECT, India**

**Terms of Reference**

Post intervention Data Collection for the Impact Evaluation

# Purpose

The purpose of this consultancy is to conduct the post-intervention (endline) household- and community-level data collection for an impact evaluation of the Total Sanitation Campaign (TSC) in state of Madhya Pradesh (MP).

# Background and Program Description

The Water and Sanitation Program (WSP), with support from the Gates Foundation, is implementing a sanitation project in India to support the Government of India’s (GoI) Total Sanitation Campaign (TSC). TSC aims to improve sanitation (end open defecation, promote school sanitation, advance environmental cleanliness) through community demand-generation activities. GoI is also providing financial incentives in terms of assistance for toilet materials to the poor and awards to Gram Panchayats (GP) upon their becoming open defecation free (ODF) and satisfying other total sanitation criteria. TSC is an ambitious program that seeks to deliver dignity, health, and welfare impacts. WSP advocates the use of Community Led Total Sanitation (CLTS) approaches to trigger the demand for sanitation and provides capacity building and training support to the local implementing agencies to ensure the effective design, implementation and monitoring of TSC.

WSP is conducting a global impact evaluation (IE) study to develop the practical knowledge needed to design sanitation programs that are effective and sustainable at-scale in poor rural areas. We hope that a well designed research study will help us learn how to scale-up a set of successfully implemented interventions, as well as effectively estimate the costs and the health and welfare impacts that can be expected from these interventions. The impact evaluation of sanitation programs is being carried out in three countries: Indonesia, India and Tanzania. In India, WSP’s impact evaluation is focused on examining the impacts of TSC in Madhya Pradesh (MP). With the approval and collaboration of the local government agencies this impact evaluation is being conducted in two Districts: Dhar and Khargone.

For the impact evaluation of TSC in MP, we have designed a randomized control trial. In each district, we randomly selected 40 GPs (multiple villages within a GP) from the sampling frame provided by the local government. Of these 40 GPs, 20 have been randomly assigned to a treatment group that receives focused TSC; the other 20 form the control group. The design includes a pre-intervention survey (baseline) and a post-intervention survey (endline) in both treatment and control GPs. The endline survey sample will be the same panel we interviewed in the baseline in 2009, and will include additional new eligible households. In the endline survey, we will interview 38 eligible households from each GP for a total sample of approximately 3000 households from both districts (38 HHs x 80 GPs). These 3000 households consist of approximately 2000 households interviewed in the baseline and approximately 1000 new, additional households that have at least 1 child who is less than 2 years of age at the time of the endline survey.

As mentioned previously, the baseline surveys in both Dhar and Khargone were completed in 2009. The implementation of TSC ended in July 2010. We have planned the endline survey to begin in January 2011 and to conclude within a maximum of 30 days. The timing of endline survey is crucial because there is only a small window of opportunity between the end of the current TSC program implementation and the beginning of new cycle of implementation. Further, the survey timing is critical because of seasonal nature of many of the disease outcomes we will be measuring in the endline.

To estimate the program impacts, we will employ a difference in difference (DID) methodology. In DID, we subtract the difference in the health outcomes between treatment and control group at the endline from the difference between these two groups at the baseline. This double difference will provide us with an estimate of the health impacts of TSC.

An additional round of follow up survey is anticipated approximately one year after the endline surveys in the same set of GPs and households to increase the robustness of our impact estimators as well as measure the sustainability of impact. This study will be contracted separately; however excellent performance in completing all data collection activities as described in this ToR may qualify the firm/consortium for single source procurement of this follow-up study in 2011-2012. *[****Note:*** *this additional study was not the part of original protocol but only at discussion/ideation stage and never pursued later]*

The impact evaluation in MP will measure a broad range of health indicators, and intensively study the developmental, social, and economic welfare impacts of TSC. Health outcomes and other measures that are explicitly planned in this study include:

- diarrheal prevalence;
- prevalence of other health outcomes such as fever, acute lower respiratory infections, and gastrointestinal diseases;
- primary health check up (chest risings, chest wall in-drawing etc);
- anthropometric measurements (head and arm circumference, height, weight, etc);
- iron deficiency / anemia using finger-prick tests;
- stool testing for parasitic infestations;
- water sample tests for E. coli and Salmonella contamination;

Some of the non-health indicators to be measured in the IE are:

- cognitive and motor development;
- school attendance, academic performance and future earnings; and
- productivity of mothers’ time for household, market and social activities
- female empowerment and security due to safer sanitation conditions

# Scope of work

The selected firm/consortium will be responsible for implementing several closely linked data collection activities and delivering quality data to the expectations of WSP principal investigators. The firm/consortium will work under the direct supervision of the project Task Team Leader in Washington DC, as well as impact evaluation principal investigators and the country program coordinators. All research and design activities (e.g., study design, design of study instruments) will be undertaken by WSP investigators. The only requirement from the survey firm will be to carry out quality data collection and ensure timely and thorough reporting.

The main data collection activities to be carried out by the firm are:

## Household surveys

A face-to-face household survey covering a sample of approximately 3000 households from 80 GPs (38 households per GP) from the aforementioned 2 districts. The household survey will have two main components: (i) household socioeconomic classification (SEC) and Knowledge Attitudes and Practices (KAP) survey; and (ii) household health survey.

### Household SEC & KAP Survey

The length of this survey is on average 80 minutes per survey. We collect detailed information on demographics, education, occupation & labor force participation, migration, household assets, water-sanitation-hygiene infrastructure, water-hygiene-food safety behaviors, children care situation, and other relevant details. The survey will have multiple respondents: the household head and each primary caretaker of a child less than 5 years old.

### Household Health survey

This survey is administered to each primary caregiver of a child less than 5 years old in the household (multiple respondents). For each child, the survey takes approximately 20 minutes. On average, households have approximately 2 children under 5 (this number is only an estimate and can change). The indicators of health measured in this survey include:

1. heights, weights, head and arm circumferences of each child under five years old. The survey firm must procure the equipment needed to make these measurements as per WHO guidelines. WSP has approved Tanita digital scale and Seca stadiometer for measurements. The survey firm should select other equipment as needed. All measurements should be repeated twice, each time by a different enumerator to ensure accuracy;
2. Iron-deficiency anemia testing using portable hematocrit blood iron test kits of household members between 6 months and 5 years of age. The provision of specialized equipment and supplies needed to measure hematocrit blood iron (hemocues, microcuvettes and lancets) will be procured and provided by WSP;
3. disease incidence for several diseases will be ascertained in a child health calendar. We will seek information on the onset and duration of the diseases, symptoms, observation of chest wall, measurement of breaths, location of treatment, expense on treatment.
4. primary check for chest rising, chest wall in-drawing, etc.

We absolutely require that the enumerators conducting health survey are thoroughly trained on all protocols and measures (including standardization (and proof thereof) on anthropometric measurements as part of their training). The team of enumerators must have relevant education and be highly experienced in health measurement surveys. We expect that the survey firm will provide special supervisors / field managers and quality assurance/quality control (QA/QC) protocols to ensure high quality in health measurements.

## Community and facility surveys

A community questionnaire will be administered to a group of 2-3 knowledgeable GP leaders. We will collect information on community facilities, government schemes and programs, environmental and health shocks, community access and connectivity and others. The community survey takes approximately 30 minutes on average to complete. Additionally, we will conduct facility surveys (15 minutes each) with 3-5 health and education facilities in or near the GP. These facilities include but are not limited to sub-PHC, PHC, schools, anganwadi, pharmacy, etc.). These surveys may have several open ended questions where we need the enumerator to explore responses through a discussion. Therefore, we recommend that (1) experienced & (1) supervisor level person be assigned to conduct these interviews.

## Microbiological survey

We will collect 1 drinking water sample from 10 households in each GP and 1 stool sample from 15 households in each GP. In addition, we will collect samples from all in use household drinking water sources. As per the baseline data, we expect 4-5 sources in total are used by households in each GP. These figures represent the minimum required sample size. The survey firm may consider over sampling to protect against loss of samples for any reason, for which the firm will be responsible for replacing. If above sample size is not met, WSP will consider the deliverable incomplete and not pay for it. All samples will be collected in sterilized containers. Water samples should be stored in ice boxes and child stool samples should be stored in Formalin solution (slightly acidic). Each sample will consist of approximately 120 ml volume. Water samples should be transported within 24 hours to a centralized laboratory in Mumbai which was used in the baseline survey as well. The child stool sample collection requires that an empty bottle be left with the households and collected the next day. The caretaker of children under 5 years old will have to be briefly trained / instructed on how to collect the child's stool sample in the bottle. Stool samples must be stored in GfK local offices and transported to WSP approved laboratory (NICED, Kolkata) every 10-12 days of field work. Along with sample collection, the enumerators will need to complete a 2-page questionnaire (10 minutes) for each sample. In this form, we note the unique GP code, household ID and source number and other information. NICED will be responsible for testing of stool samples as per WHO approved protocols using Kato-Katz and ELISA methods. GfK Mode is responsible for subcontracting with NICED to provide these services.

### Water Test requirement

The laboratory should use membrane filtration method wherein they will filter 100 mL of water and incubate using HiMedia M1466 Salmonella differential agar (salmonella and E. coli). The test results should be reported in CFU/100 mL for E. coli and Salmonella spp, both. The lab is expected to have controls in batch of tests, and re-validation of 10% results using other conventional methods. The lab should provide a technical person to train the enumerator on sample collection and join the survey firm in planning field logistics of collection and transportation. We do not require any other test that may be required as per BIS standards.

### Fecal Test requirements

Fecal samples will be collected and thoroughly mixed in formalin - a slightly acidic solution - in the field itself. It should be then transported to a central location and shipped every 10 – 12 days to NICED laboratories in Kolkata. The enumerators conducting microbial surveys must be specially trained to do a thorough and very careful job. A separate supervisor / editor should be budgeted for health surveys and microbial surveys.

# Key Tasks for Data Collection:

Key tasks required to accomplish above study components are described below.

1. **Obtain extension to the study protocol from Independent Ethics Committee (IEC), Mumbai.** This board has already approved the study design. We need to present the baseline findings and changes to the study design for the endline. We will share all documents from the baseline application to IEC with selected firm, but the firm is expected to suitably modify the materials and file application with IEC. The application process is simple but time consuming (3-4 months). Firms should quote for all fees charged by IEC to grant approval to the studies.
2. **Recruit and contract with experienced enumerators and supervisors.** Considering the complexity of the survey, minimum education and experience levels of enumerators and supervisors are required. The list of enumerators and their qualifications should be pre-approved by WSP before the main training and then qualified enumerators should be short listed after the main training in consultation with WSP; the short listing will be based on training performance including standardization on anthropometric measurements. We expect that only the selected enumerators will continue to work on the project and no enumerator will be replaced by a new enumerator who was not a part of main training. Enumerators who have not been standardized effectively on anthropometrics will not be eligible to participate in data collection efforts.
3. **List and map additional households.** WSP will share listing sheets and maps from the baseline as well as a household panel dataset from 2009 that contains 25 households per GP. For the endline survey it will be necessary for the firm to list additional households to meet the sample of 38 households per GP. For identifying new or additional households in the endline, limited listing and mapping is expected in each GP (100 Households).

If a baseline panel household has migrated, split or otherwise not available, then the firm should complete Lost Household module of the household questionnaire. The survey firm should ensure that the attrition is less than 5% from the baseline household panel, unless for reasons beyond their control (such as permanent migration). We expect that they survey firm will make up for the lost household by interviewing additional/new eligible household so that required sample size (38 HH per GP) is maintained. Completing cover page or lost household form will not be counted towards the sample size of 38 interviews per GP.

1. **Pre-test a few additional questions and flow of entire questionnaire.** All field logistics should be tested. Pre-test is expected to precede the main training, and pre-test should not be done as a part of a field practice or main training. For pre-test the survey firm should use a specialist small team consisting of experienced and senior supervisors/enumerators. This team should be a part of main training as well. All surveys and data collection procedures should be pre-tested in 50 households. The pre-test data must be entered using CSPro program provided by WSP. Data in STATA format and descriptive statistics for key variables should be submitted to WSP PIs. The firm should submit the CSPro dataset (ASCII format) to the data entry firm, Kimetrica, along with detailed comments about specific data fields that could not be accessed, specific values that could not be entered, skips that didn't work properly, etc. All pilots must be done in non-study villages in Dhar or Khargone district in a total of 50 households, preferably in at least 2 different GPs. Anthropometry and microbiology (collection of water and fecal samples) should be done in at least 10 HHs. The pilot in each GP should include the community questionnaire and rapid assessment.
2. **Translate new or modified questions into Hindi (Kruti font only).** Most of the questionnaires, field manuals and forms were translated to Hindi during the baseline survey. However, WSP will be adding new questions and modifying older ones. Any new Hindi translation should be back translated in English (as a word document) and differences reconciled.
3. **Reproduction of the questionnaires and other data collection forms in sufficient quantity.** Questionnaire will become final only post main in-class training and/or field practice. Therefore, it is essential that a field manager/researcher with good DTP skills in MS Word is available during training to make these changes immediately and direct printing operations. The key concern is the time gap between field practice and launch of surveys due to time taken in printing questionnaires. We, thus, suggest local printing (in Indore or nearby areas) press be recruited.

The questionnaires have to be formatted perfectly and printed in booklet (center pinning) format. The cover should be of a thicker paper. We should print 3 booklets for each household: (a) household SEC&KAP modules; (b) health survey modules; and (c) microbiology module. These questionnaires should always be handled as a set irrespective of one or more teams/enumerators administering them. The cover page of each booklet in a set should have a same pre-printed unique serial number. If the set has to be dismantled for ease of operations, then provisions must be made to guarantee that the set will be assembled accurately for each household. Each enumerator should also carry 10-12 A4 size laminated color flash cards to show respondents. Community survey will also be printed in a booklet format.

1. **Centralized training of selected enumerators, editors, supervisors, and field executives.** Before main training, WSP, GfK, and if possible, district TSC officers will hold a joint workshop for 1-2 days to discuss the training plan by GfK, role of WSP, and other such details. For household surveys, we require 11 days of in-class training (5 days are for health surveys), 1 day of in-class training for microbiology surveys, and 2 days of in-class training for community surveys. In-class trainings should be conducted by experienced researchers & field managers and field executives can provide support. Health survey training must be conducted by a medical professional expert in anthropometry, on-site blood test, diseases symptoms, and nutrition with good practical survey experience.

In-class sessions should be conducted in comfortable (temperature, light, food) environment, with use of transparencies &/or LCD projections (as per WSP recommendations); and interactive (role place, Q&A, etc). Each day should have a short evaluation / test which will help us select / screen good enumerators (Note, we suggest the survey firms recruit 15-20% extra enumerators to account for any drop). We may train teams for anthropometry and health survey separately so the survey firm should be prepared to make a separate room available for training.

At the end of health survey training, enumerators, editors, supervisors and executive must be *standardized* (ensuring that a measurement by different people is same with ~99% accuracy). WSP will explain and train on standardization method. The teams for health surveys will be approved by WSP on basis of these reports. GfK must arrange children between 0 to 5 years of age so that the enumerators can practice measurements and they can be standardized. We will need children for 3 days (entire day). We can discuss recruiting different sets of children for small duration so that they are not fatigued. For practice and/or standardization, GfK can partner with a local children clinic or hospital which may have steady supply of children.

After in-class trainings, we require 2 days of rigorous field training followed by a day or two of debriefing. Field practice must happen in GPs in Dhar or Khargone districts. During field practice each enumerator should interview at least 4 households; each enumerator should be accompanied at least once by a supervisor of field executive. Field practice should include transport of microbial samples to labs as well as testing by the lab. Supervisors and editors must also practice. At least 2 completed questionnaires by each enumerator should be thoroughly checked by executives, managers and researchers. WSP will provide a list of approximately 50 key questions that need to be especially checked. We also recommend field data entry of these questionnaires using laptop computers (50 key questions; 2 questionnaires per enumerator). Data entry using STATA is recommended, but Excel, FoxPro based entry will be permitted. WSP will need to review the summary d-stats prior to debriefing.

Considering that the survey firm needs to check questionnaires thoroughly, print corrected copies of questionnaires in bulk, and enter data in the field, we recommend a gap of 2-3 days between the end of field work and debriefing. Alternatively, we can conduct 1-day debriefing immediately after field practice and have a short 1-day refresher training a day before the survey launch. The firm can suggest other alternatives.

1. **Field Implementation, supervision and Management.** The survey firm should work in teams of enumerators, editors, microbial sample collectors, health survey enumerators, and supervisors. Enumerators and supervisors can play multiple roles provided it does not affect their performance. We need 100% editing of the data in the field. We also need separate supervisor for health and microbial surveys. We require at least 1 field executive to supervise on 4-5 teams continuously throughout the survey administration. Field managers should also regularly visit and monitor the teams. We also recommend that the researchers be a part of management & supervision team for at least the first several days of field work. The above are only suggestions. The firm can propose different team composition and implementation plan.

Field planning should consider the need to visit some households more than once to complete the survey. For stool sample collection, each household has to be visited twice – first to provide empty bottle for stool collection and explain the procedure to caregivers of under 5 children and then to collect the sample next day.

Informed consent is a requirement before beginning any survey. Prior to starting the work in a GP, supervisors or others should take "written" permission from Sarpanch, Deputy Sarpanch, or GP officer (permission from non-elected or non-government staff will not be accepted). These permission forms should be saved for future submission to WSP as a deliverable. Each household survey has a detailed consent form. We expect each enumerator to read it verbatim without taking any short cuts. We should take signature of the respondent as a proof of this informed consent. Supervisors’ daily report should verify that informed consent has been administered correctly.

We suggest at least 20% back checks by supervisor. The relevant section in the questionnaire should be filled completely so that we understand which households were back checked, by whom, and when. We also recommend back checks by field executives and managers as part of their routine visit. In addition, we encourage survey firms to propose additional measures to ensure data quality in field.

Daily log/reports maintained by the supervisor should include information on sample control (achieved, lost, microbial samples, migration etc.), verification that 100% editing is done, and 100% verification that health surveys are done correctly. In addition the editor should keep a log of questionnaires he edited and key/recurring errors identified on a daily basis. Considering the panel survey, the editor should have a checklist to ensure accuracy of household unique ID and member IDs as per the baseline, and verification of complete/accurate information in health survey.

We also expect that field executives, managers, and researchers visiting the field will prepare trip reports that will focus on: implementation status, field issues, data quality, key concerns, and specific activities done by them. We expect that the executives or field managers will send weekly updates to WSP on survey status (details on sample size achieved-dropped, migration, microbial sample, health measurements, etc) throughout the duration of the data collection and data preparation phases.

1. **Lab testing protocol development.** The labs selected by WSP and/or the survey firm in consultation with WSP should develop a detailed protocol for receiving the samples, testing the samples, ensuring accuracy and validity of test results, and reporting the results in a tabular format. The firm is expected to work alongside the labs to develop this protocol. The protocol should include supervision and management protocol from both lab and the survey firm.
2. **Recruit and Train experienced and expert CSPro data processing staff.** The data processing staff should include a full-time CSPro programming expert and a team of data entry operators experienced in working with CSPro. The Data processing manager and the team of data entry operators will need to be approved by WSP. Kimetrica will lead the DP Training for duration of 3 days. After data entry training is completed, the survey firm in consultation with Kimetrica and WSP will select a manager and data entry operators. Additional requirements are: (1) maximum number of keyers is capped at 10 (we prefer 5 or less); (2) a local area network linking all data entry computers; (3) the DP work must be done in GfK offices or offices nearby (no subcontracting out the DP work); (4) GfK can hire external keyers, but the DP must be in house and there must be at least one supervisory GfK person involved on a daily basis with the DP operation; and (5) adequate storage room facilities to accommodate questionnaires by clusters; clusters must be stored in numerical (according to cluster #) order.
3. **Develop Data Management Plan.** The data entry should be done in survey firms own office under direct and day-to-day supervision of approved data processing manager; no outsourcing of data processing work is permitted. The data entry must be done using the CSPro template WSP has developed. Kimetrica will provide all CSPro design and modification related support. Data management protocol will be developed by Kimetrica and will include provision for 100% double entry, data QA/QC procedures, supervision, and management details. The protocol will include a daily log of DP manager that record the key activities of the day, errors in the data and any changes in CSPro. The firm will be expected to produce weekly data processing status reports. Along with progress achieved in entering and checking the data, this report will include *field check tables* as suggested by Kimetrica. The survey firm should follow the data entry protocol developed by Kimetrica and shared with the firm.
4. **Delivery of electronic data in STATA format.** The firm should first submit the draft data to WSP PIs for checking and verification along with d-stats of key variables and codebook. Firm must address all problems in the data (multiple rounds of cleaning may be needed). To enable checking and verification of draft data by WSP, firms must submit the data in CSPro format. The firm must ensure that all variable and value labels exist and are accurate. Data should be provided at both individual and HH levels (reshaping of data). There are different questionnaire booklets for each household. Survey firm should ensure that all merging and linking of various booklets (datasets) and modules therein are possible and unique merging IDs are provided. The firm should also ensure that the data merge perfectly well with 2009 baseline panel of households and individuals.

All "other" or open ended answers should be coded whenever they exceed 3% of the responses. All variables should have variable and value labels (wherever applicable) for individual and household level datasets. The variable names should be exactly the same as the ones used in the baseline. Multiple responses to a question should be recoded to binary (0, 1) by creating new variables equal to number of multiple response possible (also known as SPSS format in DP parlance). For example, if a household can use 5 types of water sources, then the data must contain five variables, each corresponding to a specific water source type, with a value of 0 or 1 depending upon whether or not the household uses that specific water source.

The draft data should be submitted to WSP within 30 days of completion of field work. The final dataset should be given within 15 days of receiving feedback from WSP. The survey firm will provide detailed frequency tables and d-stats for approx. 100 key questions (as identified by WSP). The firm should also file report on data processing protocol and quality of delivered data.

Upon approval of the data by WSP, the firm should submit the CSPro data files to Kimetrica as specified by them. Kimetrica will further check and evaluate the data quality and ask the firm to clean the data further. The data will be considered final only after both WSP PI and Kimetrica approve it.

The data and all questionnaires should be archived for a period of 3 years from the date of delivery of the data. Before disposing of the questionnaires at the end of this period, WSP consent must be sought.

**Timeline of Tasks (Tentative)**

We expect to finalize the contract by December 2010. Our objective is to complete endline data collection no later than middle of March 2011. The following schedule of tasks is indicative and should be used to guide the survey firm to propose the workplan accordingly.

| **Key Tasks** | **Approximate Timeline** |
| --- | --- |
| Obtain extension to the study protocol from IEC | January 15, 2010. If permission is delayed for reasons beyond control of WSP and GfK, then we expect at least in principal approval from IEC |
| Recruit enumerators and supervisors | 30 December, 2010 |
| List and map additional households | 15 January, 2011 |
| Pre-test | Field work by 30 December 2010. Data reports by 15 January, 2011 |
| Translate new or modified questions | 30 December, 2010 |
| Printing questionnaires and forms | First batch within 2 days of main training. Second batch within 6 days thereafter. |
| Centralized training | 15-30 January 2011 |
| Field Implementation | 30 January – 15 March, 2011 |
| Lab testing protocol | Protocol by 15 January 2011. Draft Report and data by 15 April, 2011. Final by 30 April, 2011 |
| Recruit CSPro DP staff and DP training | 30 January, 2011 |
| Develop Data Management Plan | 15 January 2011 |
| Delivery of data | Draft Report and data by 15 April 2011. Final by 30 April, 2011 |

The schedule may change during implementation given unforeseen circumstances. In case of delays and resulting hastening of schedule, we expect the survey firms to increase the manpower and other resources to meet the deliverable deadlines. The key staff and field teams approved by WSP should remain available for the duration of the project. The approved protocols must be followed irrespective of changes in the schedule.

# Expected Deliverables

1. **Extension to study protocol from IEC.**
   1. IEC based in Mumbai had approved the study protocol in 2009. However, we need to file the report of baseline findings and any changes to the endline survey (there are minor changes in scale but not scope). More details are given on Task 1 in Section 3.
   2. We require that the firm fulfill all legal requirements for employment (e.g, insurances, worker compensation) for enumerators and/or contractors working on this project as per mandates of the Government of India and the State of Madhya Pradesh.

**Deliverable:** Letter from IEC approving study protocol until 2011 and receipt of fees paid to IEC (scanned copy is sufficient). Evidence that all staff working on this project have required insurance coverage OR assurance that no insurance coverage is required for field or research staff/consultants/free-lancers working on this project as per applicable laws of India and the State of Madhya Pradesh.

1. **Development of Study Implementation Protocol**
2. Recruitment of survey firm staff and external/internal enumerators and supervisors for the field work and data entry/processing work (see Tasks 2 & 10 above)
3. Developing field implementation, management and supervision protocol based on approved proposal and guidelines listed in Task 8, 9, 11.
4. Developing tentative field implementation schedule. This can be modified post training but we need to know how you have planned to conclude data collection within 40 days.

**Deliverables:** Protocol report that includes:

- - 1. List of enumerators, editors, supervisors, executives, data entry operators, data processing manager, field managers, and researchers who will work on the study with their qualifications and relevant experience. Assurance should be given that only these, or a subset of these people will work on the WSP study. Key staff should not change from those proposed to WSP.
    2. Detailed schedule of field work (by GPs) & data processing
    3. Procedures, Supervision and management plan details (field work and data processing)
    4. Types of daily, weekly and visit wise reports that would be generated
    5. Corrected / updated CSPro data entry template (*Needs to be further updated based on corrections during training*)

1. **List and map additional households**
2. Develop plan to list & map additional households as required. The plan should be developed based on review of listing and mapping from baseline 2009. The plan should be developed according to the approved proposal to WSP and Task 3.
3. Recruit team and develop field plans as per deliverable (2) above.
4. Prepare summary of households planned for revisit, new households listed, and number of eligible households. We recommend that the listing and mapping team make the first attempt of finding baseline panel households to save time during main survey administration.

**Deliverables:** Listing & Mapping report that includes:

- - 1. Description of procedures including teams, supervision, time taken etc.
    2. Summary of number of households listed, identified from 2009 baseline panel, eligible number of HHs
    3. All listing and mapping sheet stored in a safe location after WSP reviews and approves them.

1. **Questionnaire development**
2. As per the field protocol developed in deliverable (2) and guidance provided in Task 4, all draft questionnaires should be pretested in 50 households in study area by a small team of enumerators and executives. The errors in content, flow or logic should be identified and corrected. We will identify additional errors during main training which should be rectified immediately.
3. All changes and addition to the questionnaire post pre-testing and during main training should be made in both English and Hindi versions. Back translation of Hindi changes should be given to WSP (See Task 5 for details)
4. All questionnaires should be printed as per the requirements outlines in Task 7. The questionnaire shall be printed only after main training is conducted.

**Deliverables:**

- - 1. Pilot/Pre-test dataset in STATA format along with d-stats on key (approximately 100) questions and a narrative of the errors found or implementation issues faced.
    2. Pre-test data submitted to Kimetrica in ASCII format as per their requirement along with a detailed report (data field wise) of problems faced in entering the data in CSPro template.
    3. Soft copy of English and Hindi questionnaires in "track mode" indicating all the changes made during pre-testing and main training
    4. 20 copies of printed questionnaires should be shared with WSP staff

1. **Main Training**
2. Ensure that people listed in study implementation protocol (Deliverable 2), research and field staff proposed in the proposal, and team members involved in pilot study are present for the training session
3. Conduct training as per Task 6 described above.
4. Prepare standardization reports for all health survey team members
5. Prepare error log and correct questionnaires based on in-class and field training exercise
6. Prepare summary tables for key variables entered in the field for WSP review

**Deliverables:** Training report that includes: (i) Names of the people who participated; (ii) day wise description of the activities and enumerator evaluations; (iii) final evaluation and selection of enumerators/supervisors for the field work (Table of evaluation score and selection criteria); (iv) standardization reports of all enumerators; (v) description of field practice, narrative of key errors in questionnaire or in implementation; and (vi) dataset of key variables including their summary statistics.

1. **Field Implementation, supervision and Management Report**

This report should be developed as per the approved implementation protocol (Deliverable 2). Deliverable 2 is a plan where as this report is how things were exactly done in the field. Specifically the report can build on the protocol in terms of following:

- 1. Description of listing and mapping procedures. Provide summary data for each GP: no of households identified from 2009 panel, number of new households listed, eligible number of households, and other information as per WSP needs.
  2. Description of procedures to identify and interview households from 2009 baseline panel. Include details such as, how unique household and member IDs were maintained, if a household was unavailable, then how it was replaced, supervisor and management control over the process, attrition rate in the panel
  3. Informed consent forms signed by all GP Sarpanch or their alternatives as per requirements in Task 8. Summary table of informed consent of the households (we have 4 consents: overall survey, anemia testing, health survey, and microbial survey
  4. Describe field procedures (team size, supervision, scheduling etc), date of field work, sample size achieved
  5. Describe quality control procedures in detail: This should include (but not limited to): Back checks, spot check activities; field level editing; supervisor’s daily log; field supervision /management by senior staff; MIS tables and reports
  6. Describe microbial sample handling procedures, lab procedures, and QA/QC measures on field and in lab.

**Deliverables:** The report should be based on Deliverable 2 (implementation protocol). In addition to above narratives, it should include the following specific details:

- - 1. Listing and Mapping sheet photocopies should be given to WSP and original stored safely with the survey firm (for 3 years).
    2. Composition of a standard field survey team
    3. Survey management structure (tasks and responsibilities, communication, management)
    4. Date and team wise field work details (dates of field work are very important)
    5. Quality Control protocols and reports (summary tables and scanned/photo-copies): supervisor daily reports, editor reports, sample control sheet (hard copies and Excel data), executive-manager-researchers management reports, sample delivery and lab QAQC reports, status reports submitted weekly to WSP (please also include email print out so we know the date of delivery), and other reports as requested by WSP.

1. **Microbiology Report and Data**

A protocol for receiving the microbiological samples, testing, and reporting will be submitted as part of deliverable 2. Based on the agreed protocol we expect the selected firm to submit report and data.

The report should detail out all the procedures in the field and laboratory, results of QAQC studies in the laboratory, log and descriptions of the errors, any assumptions or correction made to the sample identification at the lab, report on status of sample received (condition, date, etc), and other such agreed items.

Because of our sample size requirement and the need to merge the microbiology data with the main household data, we will consider the data complete only when all of the following are satisfied.

- 1. Lab test results for household drinking water should be merged with the household data based on unique household id
  2. Lab test result for the stool sample must be merged with the child data based on unique household and person id. The survey firm must take enough precautions to ensure no sample is lost due to merging errors.
  3. Lab test result for water sources must be merged with community data based on unique GP id
  4. For each household water sample, data must identify the source of water and merge lab test result for source water sample with the household water test data using unique household and source ID.
  5. At the minimum, the data must include valid water test results from at least 10 households per GP and all drinking water sources in the GP (baseline average was 4.75). The data must also include stool test results from the same 10 HHs plus additional 5 HHs (total of 15 HHs per GP). The above sample size will be determined post all above merging and linking of different datasets. Any loss will be considered as complete violation of the deliverable and no payment shall be made for this deliverable.

**Deliverables:** The report should be based on Deliverable 2 (implementation protocol). In addition to above narratives, it should include the following specific details:

- - 1. Laboratory procedures
    2. Validation of results with traditional testing methods
    3. Log of sample receipt, condition, test
    4. Sample collection and transportation
    5. Log of sample collected, lost in transportation, lost at lab, valid results
    6. Data meeting above minimum standards in the format specified in Task 11 and 12

1. **Data Processing & Analysis Report and Data delivery**

The report should be based on the protocol developed as Deliverable 2.

- 1. List of people and their role in data cleaning, entry, and management.
  2. Describe the training procedures in detail
  3. Describe procedures used for data cleaning/editing, entry, and checking. Include any QAQC procedures adopted. Describe key supervision and management activities by the survey firm staff.
  4. Describe errors in the dataset. Provide code book for all variables and also compare statistics from main data and 100% double entry data, run logic checks (range checks should be automatically done while data entry by CSPro).
  5. Generate d-stat or frequency table for key variables identified by WSP (by 2 districts and total sample)
  6. Generate code list for open ended questions including "other" response code. Also ensure that value and variable labels (max 80 characters) are accurate

**Deliverable:**

- - 1. Data should be given as per the requirements in Task 12. All datasets should be in STATA format (exported files from CSPro are ok provided they have valid value and variable labels)
    2. Data for 100% double entry should be provided separately
    3. Data received from the labs (in excel or other formats) should be submitted in original form (without corrections by DP staff)
    4. Detailed report on data processing and management as per above requirement
    5. Scanned or photo-copies of daily activity/supervision reports by data manager, other management or QAQC reports. Training material should also be included.
    6. Final data should be delivered to Kimetrica in ASCII format with all related CSPro files as specified by Kimetrica during DP training.

# Schedule of Deliverables & Payments

[-------- REMOVED --------]

# Method of Selection

The method used to select the firm/consortium for this assignment will be Single Source Selection.

# Type of Contract

The contract will be a lump-sum amount paid conditional on satisfactory and timely submission of deliverables corresponding to data collection activities. Deductions will be made for failure to carry out any activity as per the agreed proposal/protocol, non availability of key proposed staff, late and/or poor quality deliverables

# Duration of Contract

The duration of the contract is expected to be from December 15, 2010 to June 30, 2011.

# Reporting Linkages and Working Arrangements

The Firm will submit deliverables and routine reporting to the Task Team Leader of the project in Washington DC, as well as to the Senior Monitoring and Evaluation Specialist for the global project and the Principal Investigator for the India impact evaluation. The Firm will work in close collaboration with a locally based country PI.

# Client Inputs

WSP will provide the following inputs to the firm/consortium:

- Standard set of questionnaires (household, biometric, intensive, longitudinal, and community) in English and Hindi (as per 2009 baseline)
- Interviewer and enumerator manuals and training materials in English and Hindi (as per 2009 baseline)
- Protocols for collection and analysis of fecal and water samples
- List of study Gram Panchayats along with panel data from 2009 baseline
- Hard copies of listing and mapping sheets available from the firm who conducted 2009 baseline survey
- A data entry program in CSPro used in 2009 baseline
- Specialized equipment and supplies for hemoglobin and microbiology testing: hemoglobin testing devices (hemocues), hemoglobin testing strips (microcuvettes), and finger pricking needles (lancets)
- Guidance from global or country PIs on study design and technical issues

# Required qualifications

The selected firm/consortium is expected to demonstrate the firm’s capability to implement the data collection effort as requested by the client and to exhibit quality standards as required by this activity. More specifically, the selected firm/consortium shall possess:

- Legal status recognized by the Government of India, enabling the organization to perform the above-mentioned tasks
- Extensive experience designing and implementing large scale household surveys in India over the past ten years.
- Strong background in public health and anthropometric/biometric measurement of children, including anemia, and experience using the HemoCue technology
- Knowledge of local formalities and customs in the implementation of household surveys, including ability to obtain all necessary permissions and permits related to the logistics of survey implementation
- Strong background in the preparation of household listings for random selection
- Good network of experienced interviewers, supervisors, and data entry clerks
- Background and expertise in both quantitative and qualitative research methodology
- Demonstrated experience in behavioral data collection
- Demonstrated experience in the design, implementation, and analysis of impact evaluations of health and sanitation programming
- Demonstrated experience in inferential statistics and econometrics

The qualifications of the proposed study team should be clearly stated in the Technical proposal and should include at least the following:

- Survey Research Manager: A person with at least 10-years experience and a proven track record of overseeing field data collection, experience managing large household surveys, developing and coordinating enumerator training, and knowledge of rural India (MP preferred). Although we don’t require any study design or analysis support, experience and expertise in health impact evaluations, sample design, econometrics will be a plus.
- Health professional: A board-certified Medical Doctor, nutritionist or public health specialist with knowledge in child development, nutrition, and expertise in collection of biometric samples (anthropometric, anemia/blood, microbial). At least 5 years of field experience in training, implementing and supervising health surveys is required.
- Field Manager(s): A person with at least 10 years experience overseeing survey data collection in the field, experience pretesting and piloting survey instruments, coordinating enumerator training, supervision and QA/QC procedures, and overall survey management.
- Field Executive (s): A person with at least 10 years experience overseeing survey data collection in the field, experience pretesting and piloting survey instruments, coordinating enumerator training, supervision and QA/QC procedures, and day-to-day survey management.
- Data Manager: A social scientist or statistician person with at least 5-years experience and a proven track record of data processing and analysis of large-scale quantitative surveys. Experience with field work, excellent knowledge of CSPro, experience of training and managing teams of data entry operators, ability to develop data management protocols, proficiency in QA/QC checks on the data, expertise in statistical and econometric analysis is required.

Additionally, the firm/consortium shall possess highly qualified staff to provide additional support (if and when required) with the following qualifications:

- Strong capacity and experience in planning and organizing large survey logistics.
- Strong capacity in data management and statistics.
- Strong knowledge in the following software: CS-Pro, and SPSS or STATA.
- Strong interpersonal skills and a team oriented spirit
- Strong commitment to quality
- Strong background in microeconomics and econometrics

Preferably, the selected firm/consortium and its key staff will also have the following qualifications:

- Strong background in microeconomics, statistics and econometrics
- Previous experience in health impact evaluation and/or
- Proven track record of handling large-scale panel data surveys and their analysis
- Proven track record of handling complex health surveys

All proposed staff may be considered as key staff and must work directly on the study. Replacement of staff or changes in level of efforts proposed may not be allowed.

***Stool Testing Details and Budget Amendment to ToR***

***on January 20, 2011***

Country-PIs for India Impact Evaluation study have identified need to modify the scope of work and deliverables required from GfK Mode Pvt Ltd. These changes have been endorsed by the Senior Evaluation Specialist.

1. **Transportation of Stool Samples to NICED Kolkata –** GfK has been contracted to collect and store stool samples in a centralized location for 2 months. This amendment will add the requirement to transport the samples to NICED lab in. Stool samples should be sent every 10-12th day by Air Cargo with proper packaging.
2. **Contracting with NICED, Kolkata –** NICED is identified as a suitable lab by WSP for stool testing. GfK Mode will be responsible for sub-contracting with NICED to provide testing services. GfK Mode will also be required to procure the testing supplies required for the stool analysis.
3. **Dropping ASQ Module –** ASQ module which was supposed to be administered to all children under 5 years will now be dropped.

## Statement of Work for Lab testing of Stool Samples

We are conducting a large scale and rigorous health impact evaluation of Total Sanitation Campaign in Madhya Pradesh. This is a randomized control trial where we measure various health and welfare related outcomes such as diarrhea prevalence, anthropometry, water quality and parasite infection. The following are the requirements for stool testing from a reputed lab. We have identified National Institute for Cholera and Enteric Diseases (NICED), Kolkata as the lab which is capable of doing the tests WSP needs.

- - - 1. We will test stool samples from 1200 under 3 years old children. Each sample must be tested using Kato-Katz method (for quantitative enumeration of Helminthes infections) AND also using ELISA method for identifying protozoan species as per WHO guideline (e.g., http://www.who.int/wormcontrol/documents/benchaids/training_manual/en/). GfK Mode will be required to buy the test kits from abroad as per the guidance of NICED, Kolkata.
      2. The samples will be collected over a period of 40 days (most likely Feb-Mar2011) and we would require the lab to provide us the test results within 2 months of receiving the first batch of samples (and 1 month after receiving the last batch). GfK will transport samples in proper packaging to NICED every 10-12 days of data collection.
      3. The lab will be responsible to train the enumerators in stool sample collection method (1 day) as well as part take in a field practice and arranging logistics (2 days). Lab will also provide or help develop manual for field data collection as well any other advice needed to make this scientifically rigorous study. GfK Mode is expected to cover the cost of travel, lodging and boarding whereas lab will most likely include their time cost as part of their quote to test stool samples.
      4. We will require the lab to enter in their data base system a short questionnaire for each stool sample such as the date of receipt, date of testing, ID and Name of the household head, GP code and name, etc. The lab is responsible to enter all this information for each sample along with the test results and provide us an excel sheet for the same. All IDs must be diligently entered because we will use these to merge with our household survey data.
      5. The lab should also provide a detailed report on the lab procedures, QAQC measures, etc at the end of the study. The lab should implement QAQC measures suggested by WHO and NABL etc boards.
      6. The laboratory should conserve and store all samples for a period of 6 months in case need for additional tests arises.
      7. The lab should provide or help us procure sample bottles, spatula, gloves and any chemical (e.g. Formalin 10%) to conserve the stool sample. The sample bottles must be adequate in size so that samples can be stored for later testing (see above point) as well as have a good sticker with the following requirement. Each sample bottle sticker will have unique identification: one will the household IS + the child ID to whom this sample belongs and another unique ID will be a pre-printed barcode/number on the bottle. The preprinted sticker on the bottle should have a unique bottle ID as well as ample space for us to write household and child IDs.
      8. The study site is 2 hours away from Indore, MP. Our teams will collect stool samples while they are conducting household interviews (the bottle will be left with the mother day before and collected with stool the next day). The survey team will be responsible for providing stool samples to NICED in Kolkata. However, we may request advice on packaging and transporting the samples safely.
      9. We have obtained ethic committee clearance from independent Ethics Committee in Mumbai (based in KEM hospital). We will not be able to send these samples out of the country for tests. Also, the lab may be required to inform of any infection in a sample as soon as they find it so that we can inform the household accordingly. If the lab has in-house ethics committee, then its timely approval is sole responsibility of the lab.

## Procedure for Lab Testing

The following is a rough guidelines to the procedure at the laboratory. NICED will follow these to the extent feasible because we are providing preserved stool samples.

- - 1. The received samples are then aliquot in 2ml microcentrifuge tubes and stored at -80^0^  c.
    2. A small part of the fresh samples (if available) or preserved sample (if possible) is subjected to microscopic analysis.
    3. The samples for microscopic analysis are subjected to three different procedures for perfect analysis of them. The procedures are as follows:-

1. A>Wet mount-Mainly use Normal saline and Iodine for the study procedure to identify *Giardia lamblia*, *Entamoeba* sp, *Blastocystis* sp, *Ascaris lumbricoides*, *Trichuris trichiura*, *Taenea* sp, *H.nana*,
2. B> Staining- The samples are stained by the following methods:-
3. Acid fast stain-To identify mainly *Cryptosporidium* sp
4. Trichrome Stain-To identify *Entamoeba* sp, *Giardia* sp.
5. C> Kato Katz-To identify mainly the helminths, like Ascaris lumbricoides, Trichuris trichiura, Shicstosoma sp, Taenia sp, Hookworm, H.nana etc
   - 1. After microscopy, all the stool samples are subjected to analysis by ELISA, by using specific Techlab kits for the detection of *Giardia lamblia*, *Entamoeba histolytica* and *Cryptosporidium* sp.
     2. Samples which are found positive in ELISA are subjected to DNA isolation by using QIAamp DNA stool Mini kit.
     3. For further confirmation , Detection PCR is done by using the isolated DNA from the ELISA positive samples followed by RFLP and sequencing for genotyping of local isolates.

| **Parasite name** | **Locus** | **Fragment size** | **Buffer** | **dNTP** | **MgCl_2_** | **Taq Polymerase** |
| --- | --- | --- | --- | --- | --- | --- |
| *Giardia lamblia* | β-giardin | 218 | 1x | 200uM | 2mM | 1.25U |
| *Cryptosporidium* sp | 18srRNA | 825 | 1x | 200uM | 3mM | 1.5U |
| *Entamoeba histolytica* | SSUrRNA | 147 | 1x | 200uM | 2mM | 1.25U |

## Deliverables

[-------- REMOVED --------]

## BUDGET

[-------- REMOVED --------]
